# miR-430 regulates zygotic mRNA during zebrafish embryogenesis

**DOI:** 10.1186/s13059-024-03197-8

**Published:** 2024-03-19

**Authors:** Danielson Baia Amaral, Rhonda Egidy, Anoja Perera, Ariel A Bazzini

**Affiliations:** 1 Stowers Institute for Medical Research, 1000 E 50th Street, Kansas City, MO 64110 USA; 2 Department of Molecular and Integrative Physiology, University of Kansas Medical Center, 3901 Rainbow Blvd, Kansas City, KS 66160 USA

**Keywords:** MicroRNA, MZT, ZGA, SLAM-seq

## Abstract

**Background:**

Early embryonic developmental programs are guided by the coordinated interplay between maternally inherited and zygotically manufactured RNAs and proteins. Although these processes happen concomitantly and affecting gene function during this period is bound to affect both pools of mRNAs, it has been challenging to study their expression dynamics separately.

**Results:**

By employing SLAM-seq, a nascent mRNA labeling transcriptomic approach, in a developmental time series we observe that over half of the early zebrafish embryo transcriptome consists of maternal-zygotic genes, emphasizing their pivotal role in early embryogenesis. We provide an hourly resolution of de novo transcriptional activation events and follow nascent mRNA trajectories, finding that most de novo transcriptional events are stable throughout this period. Additionally, by blocking microRNA-430 function, a key post transcriptional regulator during zebrafish embryogenesis, we directly show that it destabilizes hundreds of de novo transcribed mRNAs from pure zygotic as well as maternal-zygotic genes. This unveils a novel miR-430 function during embryogenesis, fine-tuning zygotic gene expression.

**Conclusion:**

These insights into zebrafish early embryo transcriptome dynamics emphasize the significance of post-transcriptional regulators in zygotic genome activation. The findings pave the way for future investigations into the coordinated interplay between transcriptional and post-transcriptional landscapes required for the establishment of animal cell identities and functions.

**Supplementary Information:**

The online version contains supplementary material available at 10.1186/s13059-024-03197-8.

## Background

Early embryonic development relies on a coordinated interplay between maternally inherited and zygotically manufactured mRNA and proteins that establish cell identities and functions. This widespread phenomenon [1], known as the maternal-to-zygotic transition (MZT), unfolds in two main acts [2] that are closely intertwined: maternal RNA decay and zygotic genome activation (ZGA). For example, Nanog loss-of-function or knock down [3–5] during zebrafish MZT affects ZGA mRNA levels directly by preventing transcriptional upregulation of its target genes and indirectly by stabilization of maternally deposited miR-430 targets, as miR-430 expression depends upon Nanog [4]. Thus, querying gene function in early embryogenesis has the potential to affect both maternal and zygotic mRNA expression.

Genes expressed during MZT can be grouped based on the extent of maternal versus zygotic mRNA expression, with Pure Maternal not being de novo transcribed, Pure Zygotic only being expressed after ZGA and Maternal-zygotic genes with mixed mRNA contribution [1, 4, 6–8]. In zebrafish, various RNA-seq-based methods have been used to assign genes in each of these three categories. Notably, single-nucleotide polymorphisms (SNPs) in different parental strains [6], pull-down of metabolically labeled mRNA followed by sequencing [7, 9], intron reads from total RNA-seq [4, 10], and analysis of splicing [11, 12], 3′UTR [13, 14] and 5′UTR [15, 16] isoforms have, to some extent, determined the maternal and zygotic components of maternal-zygotic genes. Yet, useful SNPs are only found in ~25% of protein coding genes [9], and de novo transcription from the maternal allele cannot be distinguished from their maternal mRNA fraction [6]. Similarly, pull-down experiments have weaknesses because short transcripts may be pulled down less efficiently [17] and only pulled-down mRNA (zygotic) is sequenced, obscuring analysis of total mRNA (maternal + zygotic). Additionally, several hundred genes lack introns preventing their detection using intronic reads, some introns may not accurately reflect current transcriptional events [11], and analysis of intronic reads assumes similar splicing dynamics for all genes within a sample, which may not hold true in other systems [18, 19]. Lastly, mRNA isoform analysis primarily relies on detecting mRNA isoform changes within a gene locus and therefore cannot be used to determine the zygotic contribution in maternal-zygotic genes when the mRNA isoforms remain unchanged, even if they transition from maternal to zygotic. More recently, SLAM-seq, a nascent mRNA labeling transcriptomic approach [20], has been applied to identify the genes as Pure Maternal, Maternal-zygotic, or Pure Zygotic [21]. The SLAM-seq method can identify actively transcribed mRNAs by providing embryos with a UTP analog that is incorporated into newly synthesized transcripts and is detected as T>C mutations after in vitro treatment of extracted RNA prior to sequencing, providing direct evidence of zygotic expression [20, 21]. SLAM-seq experiments in zebrafish have revealed that >60% of the 0.75 to 5.5 h post-fertilization transcriptome is comprised of maternal-zygotic genes [6, 21], ~65% of which show stable total mRNA levels yet still differ in how quickly maternal mRNA is replaced by zygotic molecules [21]. Altogether, previous work highlights that understanding how the maternal and the zygotic mRNA fractions of maternal-zygotic genes are regulated underlies >60% of MZT mRNA dynamics. Specifically, one aspect that remains largely unexplored is the extent of co-regulation of zygotic mRNA from maternal-zygotic genes and zygotic mRNA from pure zygotic genes, regardless of changes in total mRNA levels or isoforms.

Like ZGA, maternal RNA decay occurs in early (zygotic-independent) and late (zygotic-dependent) phases. A handful of factors are commonly deployed in most embryos to coordinate maternal RNA decay phases [1, 22], namely, RNA-binding proteins [23, 24], RNA modifiers [25–27], RNA secondary structures [24], codon-optimality [28–31], and microRNAs [32]. In zebrafish, one of earliest zygotically expressed genes [7, 9, 33] is a post-transcriptional regulator, miR-430, which is necessary for timely clearance of both maternal and maternal-zygotic genes [32], precise heterochromatin establishment [8], correct left-right patterning [34], and proper heart [35] and brain development [36]. Lastly, while few previously defined pure zygotic genes with miR-430 target sites showed increased expression in miR-430 locus deletion mutants compared to wild-type [37], miR-430’s role in destabilizing the zygotic mRNA fraction of maternal-zygotic and all pure zygotic genes has not been directly addressed.

Here we performed SLAM-seq during the first 8 h of zebrafish development to address some of the outstanding questions regarding transcriptomic changes during MZT and after miR-430 functional knockdown. Our results show the presence of zygotic contribution for thousands of genes previously deemed to be of maternal origin, revealing maternal-zygotic genes as the most representative class of genes expressed at the MZT, as recently reported [21]. Particularly, we determine the onset of de novo transcription genome-wide during ZGA with hourly timepoints for both pure zygotic and maternal-zygotic genes showing the temporal transcriptional activation events of ZGA. Lastly, we show that like its known role in maternal mRNA clearance, miR-430 also destabilizes hundreds of zygotically expressed transcripts revealing a new function of miR-430 during zebrafish MZT. Our findings shed light on maternal- and zygotic-only transcriptome changes during zebrafish MZT, offering novel insights and opening avenues into the study of transcriptional and post-transcriptional landscapes during early embryogenesis.

## Results

### SLAM-seq efficiently and reliably labels zygotically expressed transcripts during early zebrafish development

**Fig. 1:**
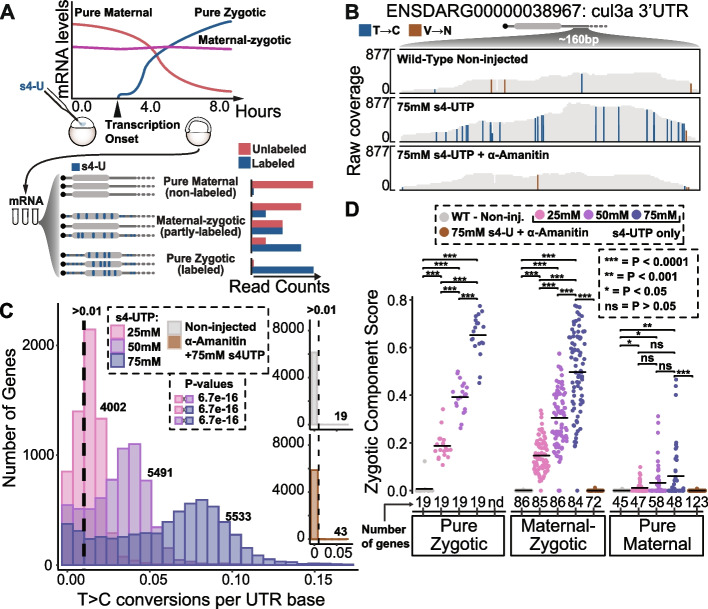
SLAM-seq T>C conversion depends on s4-UTP presence and transcription. **A** Representation of experimental set up and SLAM-seq principle applied in early zebrafish development. Injected embryos are collected at ~6h post-injection and after RNA alkylation, libraries were prepared and sequenced. s4-UTP-incorporated reads should present T>C mutations. Maternal mRNAs are expected to show background labeling (unlabeled) levels while zygotic mRNAs are expected to have T>C conversions (labeled). **B** Representative screenshots from genome tracks at cul3a 3′UTR from alignment files of SLAM-seq samples: (top) non-injected, (middle) injected with 75mM s4-UTP, and (bottom) co-injected with Alpha amanitin. T>C transitions highlighted in blue and V>N (non-T to any nucleotide) highlighted in brown. Coverage graphs on top of their respective tracks. **C** Histogram showing total number of expressed genes in each bin of mean T>C conversion rates normalized by 3′UTR base for all groups. Arbitrary cutoff of 0.01 was used to estimate number of false positives in control samples. Bonferroni corrected *p*-values highlighted from one-sided Kolmogorov-Smirnov tests. **D** Sinaplots showing mean zygotic component score (labeled reads/total reads) for different groups of genes in all SLAM-seq conditions. Number of expressed genes in each category (>5 CPMs, counts per million, nd = non-detected) is shown below each group. Bonferroni-corrected *p*-values from one-sided Wilcoxon tests as follows: *** < 0.0001, ** < 0.001, * < 0.05, ns ≥ 0.05

To define the optimal conditions for SLAM-seq in zebrafish embryos, increasing doses of s4-UTP were injected into single cell-stage embryos and development was monitored by light microscopy. No significant developmental delay was observed in the embryos injected with up to 75mM of s4-UTP, while embryos injected with 100mM were developmentally delayed at ~6 h post injection (Additional file 1: Fig. S1A, Additional file 2: Table S1). Then, to determine the labeling efficiency and specificity, SLAM-seq was performed in (i) non-injected, (ii) injected with increasing s4-UTP doses (25, 50, 75mM), and, as a negative control, (iii) 75mM of s4-UTP was co-injected with 200µg/µl of Alpha-Amanitin, a transcription inhibitor. As expected, an increase in the normalized T>C conversion rates was observed with increasing s4-UTP doses (Fig. 1B), and the T>C conversion was dependent on injection of s4-UTP (non-injected embryos showing nearly zero T>C Fig. 1B,C) as well as in zygotic transcription (co-injection of Alpha-Amanitin with 75mM of s4-UTP also shows nearly zero T>C, Fig. 1B,C). Moreover, the injections of s4-UTP did not affect global gene expression compared to non-injected embryos at ~6 h post injection (Spearman R > 0.95 and *P* < 0.05 for all pairwise comparisons, Additional file 1: Fig. S1B). Last, similar T>C conversion rate per gene distribution among biological replicates highlights the reproducibility of the SLAM-seq methodology (Additional file 1: Fig. S1C). In summary, 75mM s4-UTP injections showed the highest efficiency of labeling without affecting development or gene expression.

To validate that SLAM-seq profiles can discriminate between pure maternal and pure zygotic genes, the T-to-C conversion of previously determined exclusively maternal, exclusively zygotic, and maternal-zygotic genes were interrogated in each of the treatments. First, we determined the shared gene lists from previously published lists of pure maternal, maternal-zygotic, and pure zygotic genes [4, 6, 7, 38]. Then, the zygotic component score (ZCS), the ratio between labeled and total CPMs (read counts per million), was calculated for each gene. As expected, previously determined pure zygotic genes showed high ZCS dependent on s4-UTP injection and transcription (Fig. 1D, leftmost group, *P* < 0.0001). Maternal-zygotic genes showed a similar pattern to the pure zygotic genes and emphasize the need to untangle the zygotic component of maternal-zygotic genes (Fig. 1D, middle group, *P* < 0.0001). Lastly, pure maternal genes showed low ZCS in all conditions, although with significant increment dependent on s4-UTP and transcription (Fig. 1D, rightmost group, *P* < 0.05). Pairwise comparisons between every two s4-UTP doses injected showed that ZCS of pure zygotic and maternal-zygotic genes increases with higher s4-UTP doses (Fig. 1D, *P*< 0.0001). Last, we found no significant effects of expression levels, 3′UTR length, and T content on the ZCS of pure zygotic genes (Additional file 1: Fig. S1D, Spearman *R* ≈ 0, *P* > 0.5). These results indicate that the ZCS can accurately estimate zygotic contribution to total mRNA expression levels of each gene during early zebrafish development.

### SLAM-seq time course shows that most zebrafish MZT genes are maternal-zygotic

**Fig. 2:**
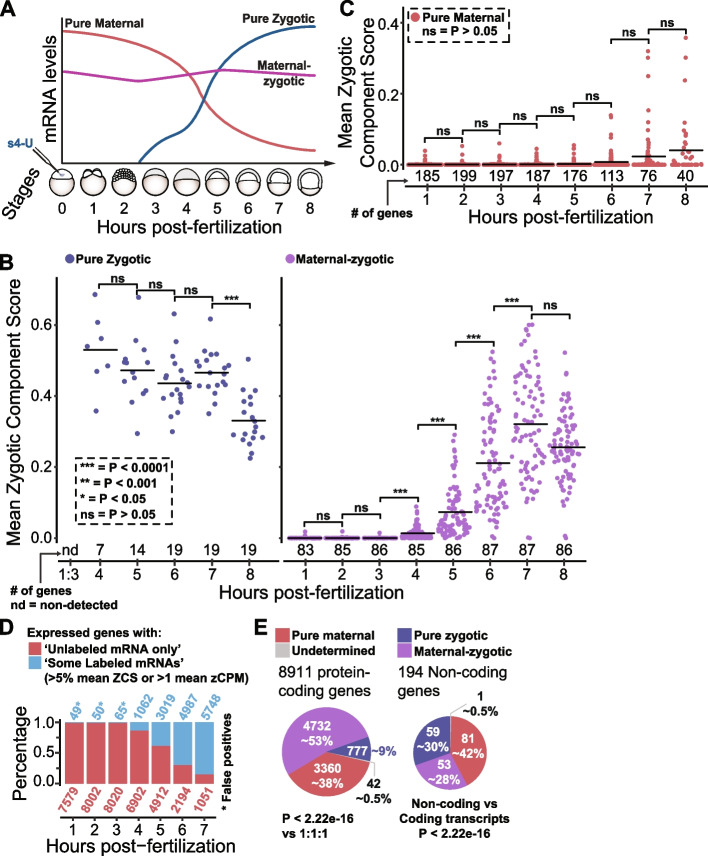
SLAM-seq shows that most genes expressed during zebrafish MZT are both maternally and zygotically expressed. **A** Representation of experimental setup and SLAM-seq time course covering 8 h of early zebrafish development, embryo developmental stages represented in the *x*-axis. **B**, **C** Sinaplots showing mean zygotic component score (labeled reads/total reads) for pure maternal, pure zygotic, and maternal-zygotic genes in all SLAM-seq time points. Number of expressed genes in each category (>5 mean CPMs, counts per million, nd = non-detected) is shown below each time point. Bonferroni corrected *p*-values from one-sided Wilcoxon tests were as follows: *** < 0.0001, ** < 0.001, * < 0.05, ns ≥ 0.05. **D** Stacked bar plots showing the percentage of expressed genes (>5 mean counts per million, CPMs) without labeled mRNAs (pure maternal) and with some level of labeling in each timepoint. Number of genes in each category colored accordingly. * False positives, as explored in Additional file 3: Fig. S2C. **E** Venn diagrams showing all protein coding genes from the nuclear genome or only non-coding transcripts, number of genes, and their percent total are shown within each diagram, *p*-values from chi-square tests are shown

To study transcriptional and post-transcriptional events during the zebrafish MZT, SLAM-seq was applied in a time series with 1-h increments comprising the first 8 h of embryo development (Fig. 2A). Several pieces of evidence indicate the quality of this dataset. First, principal component analysis shows the similarity between the replicates and the expected temporal distribution (Additional file 3: Fig. S2A). Second, there is a strong increase in labeled reads over background at 4 h post injection (Additional file 3: Fig. S2B, >18-fold when compared to 1h, Additional file 3: Fig. S2C), as expected, since labeling depends on zygotic transcription (Figure 1, transcription inhibition with Alpha-Amanitin). Third, previously defined pure zygotic, pure maternal, and maternal-zygotic genes showed the expected labelling profiles. Specifically, the previously defined zygotic group of genes show high ZCS beginning at 4 h post injection with a significant drop at 8 h, suggesting embryos may have exhausted the injected s4-UTP (Bonferroni corrected Wilcox *P* < 0.05; Fig. 2B, left side). The maternal-zygotic genes showed significant continuous increase of ZCS from 4 to 7 h (Bonferroni corrected Wilcox *P* < 0.05, Fig. 2B). The pure maternal genes displayed the lowest ZCS, never reaching significant increase (Fig. 2C, right panel, Bonferroni corrected Wilcox *P* > 0.05, Fig. 2B). These results led us to drop the 8 h timepoint for downstream analysis and further suggest that SLAM-seq estimates of zygotic contribution reproduce known changes during zebrafish MZT.

Moving beyond previously determined genes, we conducted global analysis of our SLAM-seq time course data. Genes were defined as transcribed if they had significant labeling events (>5% ZCS *or* >1 labeled CPMs) and the percentage of genes with significant transcription was plotted for each time point (Fig. 2D). As expected, the number of genes with detectable transcriptional events increased from 4 h onwards, but not all genes had a transcriptional component as we identified a group of 1051 pure maternal genes with no evidence of labeling up to 7 h post fertilization (Fig. 2D). Although we observed a relatively small number of transcribed genes from the nuclear genome before 4h, a close look at the distribution of “T” to “C” and “V” to “N” events across their 3′UTRs revealed biased “T” to “C” (consistent with naturally occurring “T” to “C” polymorphism), as opposed to randomly distributed signal observed in maternal-zygotic genes at a later timepoint (Additional file 3: Fig. S2D). Lastly, all genes with transcriptional events were classified as either maternal-zygotic (transcribed genes that are also expressed at 1–3h) or pure zygotic (transcribed genes expressed only after 3h). As previously shown [1, 2, 4, 6, 7, 17] and recently published [21], the three classes of genes are not evenly distributed (chi-square, *P* < 2.22e−16) with most of the protein-coding transcriptome being maternal-zygotic (Fig. 2E). Interestingly, the long non-coding transcripts showed a different distribution compared to the coding transcripts, containing a larger proportion of pure zygotic transcripts (chi-square, *P* < 2.22e−16). Together, these results indicate that SLAM-seq captures the switch from a maternal to a zygotic-driven control of gene expression, providing direct evidence of mRNA fractions (coding and non-coding transcripts) that are maternally provided and zygotically expressed, and show that most genes expressed throughout zebrafish MZT are maternal-zygotic.

### SLAM-seq resolved on an hourly basis uncovers temporarily grouped zygotic transcription onset

**Fig. 3:**
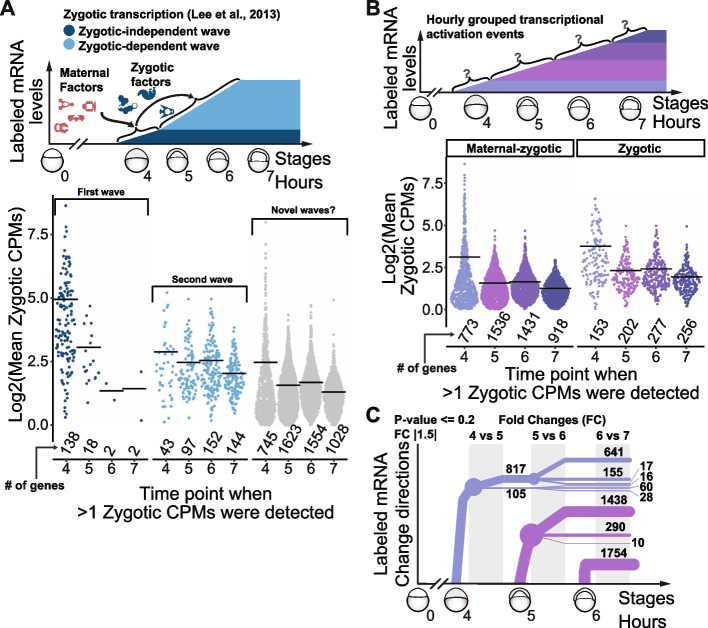
SLAM-seq reveals timely coordination of transcriptional onset of genes during zebrafish maternal-to-zygotic transition. **A** Schematic representation of previously determined transcription waves depended and in-depended on zygotic products during zebrafish development (top, Lee et al., 2013). Sinaplots showing the mean zygotic counts per million (zCPMs) for each hour, only once per gene (at the time >1 zCPM is detected) for waves depended and in-depended on zygotic products, plus all other detected transcribed genes (gray). Black bars represent mean values for each group, and total number of genes in each timepoint is shown below each group. **B** Schematic representation of hourly grouped transcription output events during zebrafish development, regardless of when within each hour they were activated (top). Sinaplots showing the mean zygotic counts per million (zCPMs) for each hour, only once per gene (at the time >1 zCPM is detected) for maternal-zygotic and zygotic genes (Fig. 2D). Black bars represent mean values for each group, and total number of genes in each timepoint is shown below each group. **C** Dendrogram showing the expression dynamics of transcriptional output throughout zebrafish development. Positive line slope indicates positive fold changes (fold change ≥ log2(1.5), adjusted *p*-value ≤ 0.2), negative slope indicates negative fold changes (fold change ≤ -log2(1.5), adjusted *p*-value ≤ 0.2), flat lines (slope = 0) indicate all other genes (> −log2(1.5) and < log2(1.5) or adjusted *p*-value > 0.2)

To explore the dynamics of zygotic transcription throughout zebrafish MZT, the first transcriptional time where each zygotic and maternal-zygotic gene shows > 1 labeled CPMs was analyzed as a function of time. Two approaches were taken: First, as a proof of concept, we analyzed the labeled CPMs of zygotically expressed genes previously shown to be dependent or independent of zygotic proteins. These two groups were created after blocking splicing by injection of U1U2 morpholino and their zygotic expression was based on intron reads using rRNA depleted transcriptome-wide studies [4]. As expected, the genes expressed independent of zygotic proteins showed significant labeled CPMs earlier than the zygotic-dependent group evidencing two consecutive waves (Fig. 3A). Yet, many genes are left out of this analysis as they were not previously determined as zygotic-dependent or -independent (Fig. 3A, gray dots), which reflects the fact that most of the transcribed genes may not follow an exclusive bimodal type of regulation. Therefore, in a second approach, all maternal-zygotic and zygotic genes (as defined in Fig. 2D) were grouped by their transcriptional activation timepoint (>1 labeled CPMs in at least two replicates), which we identified as hourly zygotic transcriptional activation events (Fig. 3B). Interestingly, this novel hourly grouped transcriptional events show many maternal-zygotic genes with similar labeled CPMs as pure zygotic genes. Additionally, for most labeling events, once a gene is zygotically transcribed (i.e., >1 labeled CPMs), it tends to keep stable labeling levels or upregulate them (Fig. 3C). Lastly, GO term enrichment analysis shows significant enrichment for terms related to RNA life cycle, transcription, and Pol-2 regulation, among others (Additional file 4: Fig. S3A, *P* < 0.05). Assembled, these results reveal novel temporal transcriptionally active groups identified using hourly timepoints, which are continuously maintained, and drive expression of genes related to transcriptional and post-transcriptional regulation.

### Inhibition of miR-430 function reveal potential underestimation of transcriptional activity by SLAM-seq

**Fig. 4:**
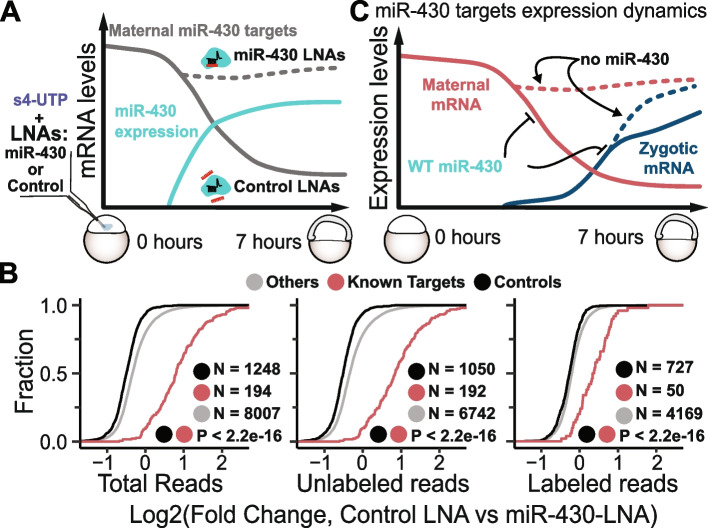
SLAM-seq reveals that known microRNA-430 regulates zygotic mRNA of its targets. **A** Schematic representation of experimental setup of SLAM-seq in LNA-injected embryos. Embryos injected with s4-UTP were either co-injected with miR-430-LNA or Control LNA, then collected at ~6h post-injection in six replicas. While miR-430-LNA reduces miR-430 function by competing with targets, control LNA does not affect miR-430 function. **B** Cumulative distributions of total (right side); unlabeled (maternal, middle) and labeled (zygotic, left) mRNA Log2(fold changes) between Control LNA and miR-430-LNA, highlighting previously determined miR-430 non-target controls (black), and miR-430 targets (known targets, red, [28]) and all other genes (gray). Number of genes in each category is shown, along with *p*-values from one-sided Kolmogorov-Smirnov pairwise comparison tests. **C** Schematic representation of main findings from this figure. Decreasing miR-430 function (miR-430-LNA) leads to increment in labeled reads of miR-430 targets when compared to control LNA. Thus, besides pure maternal mRNA clearance, miR-430 post-transcriptionally regulates the zygotic fraction of its targets. This regulation is alleviated when the microRNA function is decreased

The incorporation of s4-UTP measured by SLAM-seq has been taken as way to estimate transcription [39]; however, this technique relies on the quantification of s4-UTP incorporation in fully processed mature mRNAs. Hence, the level of mature mRNA depends on transcription as well on in mRNA stability [31]. During zebrafish early development, a zygotically expressed microRNA, miR-430, affects mRNA stability and translation of several maternally provided mRNAs [32, 37, 40]. Therefore, to interrogate the “transcription” component of miR-430 targets in the absence of miR-430 activity, SLAM-seq was performed in the presence and absence of miR-430 function. Specifically, tiny Locked Nucleic Acid (LNA) complementary to the miR-430 target sites (miR-430-LNA, [41]), or to a mismatch sequence (control LNA), were injected at one cell stage and SLAM-seq was performed at shield stage (~6 h post-injection, Fig. 4A). Multiple lines of evidence support both the feasibility and efficacy of this miR-430 loss-of-function [4]. First, non-significant changes in development of embryos injected with LNA control vs miR430-LNA were observed when co-injected with 50mM s4-UTP after 6 h post-injection, although higher doses increased death (Additional file 5: Fig. S4A). Second, previously reported miR-430 targets ( [29, 32, 40], see “ Methods”) showed significant upregulation in embryos injected with miR430-LNA compared to control LNA and non-injected embryos, as shown by qRT-PCR (Additional file 5: Fig. S4B, *P* < 0.05 [34], not observed in LNA controls and non-injected embryos (Additional file 5: Fig. S4C). Fourth, previously reported miR-430 targets showed higher total and unlabeled (Maternal) mRNA fold changes in miR430-LNA with respect to the LNA-control embryos compared to a control group of genes lacking miR-430 target site [40]; Fig. 4B, left-side and middle graphs, red vs black). Surprisingly, the known targets (50 out of 194, which showed > 2 labeled CPMs) also displayed a higher fold change of labeled mRNA in the miR430-LNA with respect to the LNA-control embryos (Fig. 4B, right-side graph, red vs black), indicating that their newly transcribed mRNAs are also under miR-430 post-transcriptional regulation. These results strongly suggest that LNA injection efficiently decreases miR-430 function during early zebrafish development and that in the absence of miR-430 function there is a de-repression of both maternal and zygotic fractions of its known targets (Fig. 4D). Thus, for transcripts under strong post-transcriptional regulation such as miR-430 targets during MZT, the “transcriptional” rates inferred from labeled SLAM-seq reads may be underestimated, due to the relatively strong post-transcriptional decay of their labeled mRNAs themselves.

### SLAM-seq reveals a wider repertoire of maternal-zygotic and pure zygotic transcripts under miR-430 regulation

**Fig. 5:**
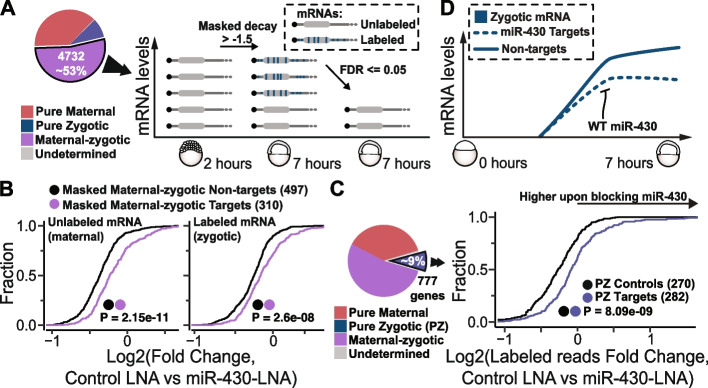
SLAM-seq reveals hundreds of zygotically expressed transcripts post-transcriptionally regulated by miR-430. **A** Schematic representation of finding maternal-zygotic miR-430 targets whose decay is masked by de novo transcription. Maternal-zygotic genes (excluding known miR-430 targets) with non-significant decay at the total mRNA level between 2 and 7 h post-fertilization (foldchanges > −1.5) but with a significant decrease (adjusted *p*-values ≤ 0.05, fold change < 0) in total mRNA vs unlabeled mRNA at 7 h were selected. **B** Cumulative distributions of total vs unlabeled mRNA at 7h (**B**, left), Control LNA vs miR-430-LNA unlabeled (maternal, **B**, middle) and labeled (zygotic, **B**, right) mRNA Log2(fold changes), genes whose decay is masked by de novo transcription that contain a miR-430 target site in their 3′UTR (violet) or without miR-430 target site (black). Number of genes in each category is shown, along with *p*-values from Kolmogorov-Smirnov pairwise comparison tests. **C** Pie chart highlighting the genes used in the analysis. Cumulative distributions of Control LNA vs miR-430-LNA labeled (zygotic, C, middle) mRNA Log2(fold changes), for pure zygotic putative miR-430 targets (blue) and non-targets (black). Number of genes in each category is shown, along with *p*-values from Kolmogorov-Smirnov pairwise comparison tests. **D** Schematic representation of main findings from this figure. miR-430 post-transcriptionally regulates the zygotic mRNAs. During ZGA newly expressed mRNA targets of miR-430 show less stability than those that do not

After our previous findings (Fig. 4), we wondered whether miR-430 destabilization activity might be masked by zygotic expression of potential miR-430 targets. We identified 310 maternal-zygotic genes containing a binding site for miR-430 in their 3′UTR that are not efficiently downregulated at 7 h when compared to 2 h at the total mRNA level. However, those genes show significant (adjusted *P*-values ≤ 0.05) decrease in unlabeled vs total mRNA fold changes at 7 h (Fig. 5A), indicating that the maternal (unlabeled) mRNA is downregulated. Therefore, we termed masked decay to the differences at the RNA level between total and unlabeled reads. Interestingly, the group of potential miR-430 targets that are not changing at the total mRNA level shows higher unlabeled reads fold changes in miR-430 LNA with respect to control LNA than a control set of genes (masked decay non-targets, Fig. 5B, leftmost plot, *P* = 2.15e−11). These results indicate that their maternal component is degraded by miR-430. Interestingly, as we showed for the known targets (Fig. 4), they display higher labeled mRNA fold changes in miR-430 LNA with respect to control LNA than control transcripts (Fig. 5B, *P*= 2.6e−08). Hence, these results reveal that miR-430 regulation masked by the zygotic contribution and that the zygotic contribution of maternal-and-zygotic genes can be regulated by miR430.

Last, we investigated whether pure zygotic genes (as defined in Figure 2) that contain miR-430 target sites in the 3′UTR can be regulated by miR-430 as the previously discussed maternal-zygotic targets (Fig. 5B). The labeling levels of zygotic genes containing miR-430 target site in the 3′UTR were analyzed in the presence and absence of miR-430 function (Fig. 5A). There are 282 pure zygotic genes with at least one miR-430 binding site in their 3′UTR. These pure zygotic miR-430 targets show higher labeled (zygotic) mRNA fold changes in miR430-LNA with respect to LNA-control than a control group of zygotic genes lacking miR-430 target site (expressed at similar levels in control LNA embryos, Fig. 5C). These results provide direct evidence that miR-430 targets pure zygotic mRNAs. Therefore, our results assembled show that, as previously suggested [32, 37], miR-430 post-transcriptionally regulates mRNAs in all sides of the maternal-zygotic spectrum (pure maternal, maternal-zygotic, and pure zygotic) and shows that the “transcription” component measured by SLAM-seq labeled reads is the results of transcription and post-transcriptional mRNA regulation.

## Discussion

Applying SLAM-seq to zebrafish embryos allowed us to (i) find that >70% of all mRNAs expressed (both, coding and long non-coding transcripts) during zebrafish MZT are maternally provided (highlighting the importance of post-transcriptional regulators); (ii) show that >50% of MZT’s transcriptome is composed of maternal-zygotic genes (emphasizing the need for tools to understand their maternal and zygotic component separately); (iii) show that ~11% of the genes, roughly one thousand, are stable pure maternally provided genes that persist up to the beginning of gastrulation (spotlighting the long-lasting maternal control over embryo development [42]). These findings agree with previous [1, 2, 4, 6, 7, 17] and recent [21] reports in zebrafish MZT and provide transcriptome-wide datasets that are valuable resources to address standing questions in the field.

Our SLAM-seq time course dissects the second main aspect of the MZT, ZGA [17, 43–45], determining temporal transcriptional activation events using hourly timepoints (Fig. 3A,B). Zebrafish genome awakening has also been explored in terms of chromatin dynamics and 3D structure, including accessibility, histone post-translational, and DNA modifications [46–56], yet their final reflections on mRNA levels have been obscured by the challenge of disentangling the zygotic contribution from total mRNA levels in a timeseries. Here the newly synthesized transcript dynamics were analyzed separately from their total mRNA levels, revealing that most of newly transcriptional output events are either maintained or increased (Fig. 3C). Thus, most of the zygotically expressed mRNAs are either continuously transcribed and/or post-transcriptionally stabilized. Still, whether the hourly grouped transcriptional events occur discretely or if they stem from gradual increases in transcriptional activity over shorter time intervals remains to be addressed. Likewise, if their onsets are fully independent of each other or are controlled by common underlying transcriptional/post-transcriptional factors in a cascade fashion needs further investigation. The last hypothesis requires applying SLAM-seq in combination with gene perturbation approaches to dissect the role of candidate genes from each group in affecting the following one transcription onset. For example, CRISPR-Cas13d mRNA knock-down of Nanog in zebrafish embryos recapitulates the molecular and developmental phenotypes of morpholinos targeting Nanog [3]. So, to dissect how loss of Nanog affects the hourly grouped transcriptional activation events, SLAM-seq might be coupled with CRISPR-Cas13d system [57]. In sum, our hourly grouped transcriptional events comprise a useful parameter to access how factors affect timely regulated zebrafish ZGA.

Besides their known role in post-transcriptional gene silencing, microRNAs have also been implicated in transcriptional adaptation (aka genetic compensation, [58]), where mRNA decay unleashes transcriptional upregulation of genes with similar nucleotide sequence [59, 60]. Interestingly, we found that miR-430 elicits maternal mRNA decay not only of pure maternal, but also hundreds of maternal-zygotic genes. Considering the results from previous sections (Figs. 4 and 5), there seems to be no global transcriptional adaptation response for maternal-zygotic miR-430 targets, at least intra locus, as once miR-430 is blocked there is an increment in their labeling, rather than a decrease (Fig. 4). However, whether specific genes and/or other MZT mRNA decay factors, such codon optimality or RNA modification, may illicit such response remains to be addressed.

miR-430 plays a crucial role in decay and translation repression of maternal mRNA during zebrafish MZT [32, 37, 40, 41]. Notably, few previously defined pure zygotic genes have been identified to be upregulated in the absence of miR-430 activity [37]. By utilizing SLAM-seq to distinguish between maternal and zygotic components, our study presents the first direct evidence that miR-430 also post-transcriptionally regulates the zygotic fraction of maternal-zygotic genes during zebrafish MZT. As for pure zygotic genes, our SLAM-seq data also provides a genome-wide evidence that miR-430 post-transcriptionally affect their mRNA accumulation, which is a different perspective than previously alluded [37]. Although miR-430 also regulates genes in specific tissues for proper organogenesis, presumably through targeting purely zygotic genes [35, 36], our results underscore its function during earlier developmental stages. This supports a previously alluded role for microRNAs on regulating mRNAs of genes that are concurrently and continuously de novo transcribed [32, 61, 62]: Keeping zygotic mRNAs accumulation at bay in time and/or space during MZT. As an example, *left2* and *stq*, which regulate embryo left-right asymmetry and are targeted by miR-430 [34], are also pure zygotic genes (Additional file 6: Table S2).

During zebrafish MZT, pioneer transcription factors regulate transcription activation widely and then cooperatively, followed by timely and/or spatially restricted ones [63, 64]. Similarly, it is likely that many general post-transcriptional regulators, such as codon-optimality, 5′ and 3′UTR structure as well as poly-A tail length, would be followed by cooperative/disruptive interactions [29] with more specialized ones to regulate embryogenesis. For example, miR-430 zygote-wide expression [37] may independently fine-tune the levels of expression of multiple zygotic mRNAs in space and/or time. Therefore, spatial information on post-transcriptional regulators, and their targets, will elucidate what their contribution is to regulate zygotic transcripts during animal development. In sum, the degradation of zygotic mRNA might provide a different view of awaking the genome, and rather to have a cascade footprint, where one transcription factor turns on specific transcription factors that will control other genes’ transcription [1]. It can be proposed that the genome needs to become transcriptionally active globally and post-transcriptional regulation fine tunes which RNAs should not be accumulated. Yet, a crucial question remains unanswered: to what extent do post-transcriptional regulators silence the newly activated zygotic transcriptome, preventing de novo transcriptional events from translating into functional proteins?

While the 4-SU labeling signal has been used as an estimate of zygotic contribution in different model systems (Fig. 2; [21, 65]), it is noteworthy to mention that they may not quantitatively convey transcription levels. We found that miR-430 targets display a higher “transcription” in the lack of miR-430 activity, likely because it is masked by miR-430 destabilization of mature labeled mRNAs in the LNA-controls, and presumably in wild-type conditions. While miR-430 is a strong negative regulator of mRNA stability, we expect the opposite to be true for positive regulators, such as m5C targets and/or transcripts enriched in optimal codons [28]. Here, miR-430 serves as an example that the “transcription” component estimated by SLAM-seq labeled reads may be miss-estimated for genes whose mRNA stability is strongly affected by post-transcriptional mechanisms. Thus, we suggest careful interpretation of results from SLAM-seq, as post-transcriptional regulation is a common feature of diverse cell types and biological processes.

## Conclusions

SLAM-seq enables the genome-wide discernment of the zygotic and maternal components for every gene. This innovative approach offers a novel avenue for investigating the dynamics of gene expression during the earliest stages of embryogenesis. Here, we determined hourly sampled zygote transcriptional activation events, laying the groundwork for future investigations into common features among genes within each hourly sampled transcriptional output group, as well as the identification of targets for pioneer transcription factors in a time-dependent manner. Additionally, we dissected miR-430 role in post-transcriptionally regulating maternal-zygotic genes and provided supporting evidence for the microRNA role in globally tuning the pure zygotic transcriptome during zebrafish MZT.

Our research unveils novel insights into the dynamics of maternal and zygotic transcripts during earliest stages of animal development, highlighting both their similarities and differences. These findings not only offer fresh perspectives on but also open new horizons to investigating the intricate transcriptional and post-transcriptional alterations that take place in the initial phases of animal development.

## Methods

### Zebrafish work

Zebrafish embryos were collected from natural breeding, with random mating parents (cross from hybrid parents [AB-TU]x[TL-TLF] backgrounds, 6–24 months old). Six mating tanks were set with 4 fish each, 1:1 sex ratio, fish were allowed to breed for 5–10 min. For each set of experiments both control and test embryos were kept at 28.5°C in 0.5x Embryo Media. All stages cited in this work follow Kimmel [66], unless stated otherwise.

### Injections

All single-cell embryos were chemically dechorionated by immersion in Pronase from *Streptomyces griseus* (Roche), at final concentration of 1mg/ml for 5 min. All injections were composed of ~1000pL per embryo, and injection volume was estimated in a drop of mineral oil under the scope. For SLAM-seq experiments, involving s4-UTP, all embryos were injected under red light and kept in the dark. For Figure 1 conditions, embryos were injected with 25, 50, 75, and 100mM of s4-UTP in Nuclease-free water, controls included (1) non-injected embryos and (2) Alpha-Amanitin (200µg/µl) + 75mM s4-UTP injected embryos. For the SLAM-seq time course, embryos were injected with 75mM and collected at 4 cells and 64 cells, plus 5 timepoints, hourly after 64 cells. For LNAs, embryos were injected with 50mM of s4-UTP, plus either LNA-Mismatch (5′-TGACATCT-3′) or LNA-430 (5′-TAGCACTT-3′) at 20µM, non-injected controls were also included. Injected embryos were then collected once LNA-Mismatch reached shield stage. For all injections, only the conditions reported in the figures were sent for their respective experiments, these being RNA sequencing, qPCR, and staging/counting/photographing. All embryo images were obtained with LEICA DFC9000GT and Leica Application Suite X v3.7.4. Scale bars were obtained from metadata file in FIJI.

### Embryo collection and RNA extraction

All embryos were collected in tubes covered from light containing 500µl of Trizol™, vortexed for 30–60 s, immediately frozen in dry ice, and stored at −70°C for no more than a week prior to being processed. There were 15 embryos per replicate for SLAM-seq Figure 1 conditions and time course, while for LNA injections, 30 embryos were collected per replicate. All the SLAM-seq samples were covered with foil or put on tubes that prevent low-wavelength light to pass through (to prevent s4-UTP crosslinking). Samples that were solely used for qPCR had 10 embryos collected per replicate, unless stated otherwise.

### RT-qPCR

After RNA extraction, cDNA was synthesized from ~2.5µg total RNA using SuperScript™ IV First-Strand Synthesis System, following the manufacturer’s protocol. To set up the real-time qPCR reactions, 1:8 cDNA dilutions were used, with forward and reverse primers at 10 μM each in a 10 μL reaction volume with SyberGreen Master MIX, automated by Freedom EVO® PCR workstation (Tecan) and ran in QuantStudio 5 Thermocycler (Applied Biosystem). PCR cycling profiles had a denaturing step at 95 °C for 30 s, followed by 40 cycles at 95 °C and 60 °C for 10 and 30 s, respectively. 2^-Delta-Delta CT analysis was performed in all conditions, as described by Livak and Schmittgen [67], all genes were normalized to pop5 CT values. The following primers were used:

- pop5_forward <AGCTCCAACGAATGCTCCTA>.
- pop5_reverse <ACTTCCATCAGTTTCCTCTCCT>.
- mrpl13_forward <TCCAGAGAAGGCGATGTGTT>.
- mrpl13_reverse <ATCGCCTCTATGTGTGCAGT>.
- gapdhs_forward <GCTGGCATCTCCCTCAATGA>.
- gapdhs_reverse <AATGGTCTGGCTTTTCTGCG>.
- smarca2_forward <CCCCTTCAACAGACCCCAG>.
- smarca2_reverse <AGCCGAGATCAACAGCTTCT>.
- me3_forward <TGGAGGAAGTGGTGGAAACC>.
- me3_reverse <TTGCTTGTGGGATTGCTGAG>.
- snx1a_forward <AGTGTGTGTGGAGCTGAAGA>.
- snx1a_reverse <TTTTGAATGGCCGGTTGTGT>.
- pora_forward <TCGAAGCGTTGTGGTGTTTT>.
- pora_reverse <GCAGAACACAGCCATCGATT>.
- sod1_forward <CGCACTTCAACCCTCATGAC>.
- sod1_reverse <TTTTGCAACACCACTGGCA>.

### Quantification and statistical analysis

Sample size was not predetermined by any statistical methods, none of the experiments were randomized, and no blind test was performed neither during experimental procedures nor during outcome assessment. No data were excluded from the analysis unless specified otherwise.

### Previously published gene lists

The overlapping of previously published gene lists [4, 6, 7, 38] was used to classify genes as Pure Zygotic, Pure Maternal, and Maternal-zygotic on Figure 1, Supplementary Fig. 1, and Figure 2B. Additionally, a list of known miR-430 targets (genes < −2 Log2[Fold Change lower in RNA-seq from WT to Dicer KO embryos] [40]) was used for Figures 4.

### 3′UTR usage and miR-430 binding site prediction

All zebrafish 3′UTRs were grouped by Ensembl gene id at 2 and 7h, the 3′UTR with the highest proportion of mapped reads (highest expressed at each time point, typically >50%) was assigned as the main isoform, and all reads from that gene id were re-assigned to that 3′UTR. DNA sequences from each main 3′UTR were retrieved from Ensembl using Biomart, then, miR-430 binding sites were searched in each individual 3′UTR sequence using a simple pattern finder for all different types, 6-mer (GCACTT), s6mer-O (AGCACT), 7mer-A1 (GCACTTA), 7mer-M8 (AGCACTT), and 8-mer (AGCACTTA). When sites were overlapping, only the longest one was kept.

### Library prep and sequencing

3′-end mRNA sequencing libraries were generated, according to the manufacturer’s instructions, from 500 ng of S4U alkylated total RNA, using the QuantSeq 3′ mRNA‐Seq Library Prep Kit for Illumina (FWD) with 24 reactions (Lexogen GmbH, cat. no. 015.24) or the QuantSeq 3′ mRNA-Seq Library Prep Kit for Illumina UDI Bundle (FWD, Lexogen GmbH, cat. no. 144.96). ERCC-92 RNA spike-ins were added at equal final molarity to all samples. For PCR amplification, 12 cycles were used with the Lexogen i7 6-nt Index Set or 13 cycles with the Lexogen Unique Dual Index (UDI) 12-nt Index Set B1. The resulting libraries were checked for quality and quantity using the Qubit Fluorometer (Life Technologies) and the Bioanalyzer (Agilent). Libraries were pooled, re-quantified, and sequenced as 75-bp single reads on the NextSeq 500 (Illumina) for the single 6-nt indexed libraries (Data from Figure 1 and Supplementary Fig. 1) or as 100-bp single reads (Data from all other figures) on the NextSeq 2000 (Illumina) for the dual 12-nt indexed libraries, each targeting 30–50 million reads per sample. Following sequencing, Illumina Primary Analysis RTA and bcl2fastq2 were run to demultiplex reads for all libraries and generate FASTQ files.

### SLAM-seq data analysis

Zebrafish ENS102 annotation for protein coding genes 3′UTRs and non-coding genes coordinates were used as reference to get reads per 3′UTR or non-coding gene. Adapters were cut from FASTQ files using TRIMGALORE [68] with the following parameters: <-a AGATCGGAAGAGCACACGTCTGAACTCCAGTCAC --length 30>. Then, all files were mapped to danRer11 genome using the slamdunk 0.4.3. [20]. Reads that contain at least 2 T>C conversions were deemed labeled. While data for figure 1 did not use any SNP calling for analysis, all the other samples used a vcf that was obtained from merging all replicas from both Alpha-Amanitin+75mM s4-UTP and non-injected embryos and calling variants from that single bam file with: <slamdunk snp -f 0.15 -c7>. Later, all raw read counts were collapsed by Ensembl gene IDs (all 3′UTRs counts summed by gene ID). Read counts per million (cpm) were obtained using EdgeR [69] after lowly expressed genes were removed (10 reads in at least some samples, 20 reads in at least one condition, half of samples per condition must have >0 reads) and library sizes re-adjusted (spike-ins ERCC-92 were used as scaling factors for library size). Scaling factors and library sizes from total read counts were also used for both unlabeled and labeled read counts per million and fold changes. EdgeR was used to obtain fold changes and FDR for total, labeled, and unlabeled reads. One replicate from both 2- and 6-h time points was discarded from the analysis due to their extremely low sequence depth and spike-ins over-representation, respectively.

### Gene ontology term analysis

The Gene Ontology Enrichment analysis was conducted on EdgeR using ClusterProfiler [70], with each unranked lists of genes from each hourly grouped transcriptional activation events (Figure 3, Additional file 6: Table S2) used individually and the background genes being all genes expressed (>5CPMs) at their respective hour.

### Supplementary Information

**Additional file 1:** **Fig. S1.** Zebrafish embryos development after s4-UTP injections, SLAM-seq/Quant-seq transcriptomic comparisons of wild-type, α-Amanitin injected embryos to regular Poly-A enriched RNA-seq. SLAM-seq ‘T’ to ‘C’ rates reproducibility and correlation between labeled reads percentage and UTR length, ‘T’ content or RNA level.

**Additional file 2:** **Table S1.** Comma-separated values, <.csv>; Total raw read and labeled read counts for all experiments and samples, including spike-ins. Rows represent different 3′UTR isoforms (for coding genes) or full-length transcript (non-coding genes). Columns represent: A, Ensembl gene IDs by itself (non-coding transcripts) + 3′UTR isoforms (coding transcripts), or ERCC IDs (spike ins); B, Chromosome name; C, Genomic start of feature; D, Genomic end of feature; E; DNA strand of feature; F, Number of Ts within feature; H-AO, Total raw read counts; AP-BW, Labeled read counts. Column names from H to BW contain informative names for stages in hours and replicates or treatment condition (LNA_Mis = control LNA, LNA430 = miR-430 LNA).

**Additional file 3:** **Fig. S2.** SLAM-seq replicate and time course grouping analysis, detection of increasing labeled reads percentage, false positive labeling events and pipeline for gene class identification (Pure Maternal, Maternal-zygotic, Pure Zygotic).

**Additional file 4:** **Fig. S3.** Results from Gene Ontology enrichment analysis for hourly grouped transcriptional activation events.

**Additional file 5:** **Fig. S4.** Titration of s4-UTP and LNAs injection, assessment of miR-430 targets de-repression at shield stage and phenotype at one day post-fertilization.

**Additional file 6:** **Table S2.** Comma-separated values, <.csv>; Coding and non-coding gene’s MZT class, hourly grouped transcriptional activation events and miR-430 targets. Each row represents a single gene. Columns represent: A, Ensembl gene IDs; B, Whether or not genes are annotated as coding or non-coding; C, MZT gene class as explained in Additional file 3: Fig. S2; D, To which of the hourly groups of transcriptional activation events the gene was assigned, if any; E; Highest expressed 3′UTR isoform used for miR-430 target prediction (see methods); F-J, Number and types of miR-430 binding sites found in the 3′UTR (see methods); K, Whether or not this gene has been previously deemed a miR-430 target, a control gene, a new putative target (putative_target; this study), or none of the previous (others).

**Additional file 7.** Peer review history.

## Data Availability

Sequencing data have been deposited in the NCBI Gene Expression Omnibus, GSE247935 [71]. Additionally, all raw read counts from all SLAM-seqs and maternal-zygotic gene classes of miR-430 targets and non-targets are provided in Additional file 2: Table S1 and Additional file 6: Table S2. All remaining original non-NGS data is available at the Stowers Original Data Repository at <http://www.stowers.org/research/publications/libpb-2403>. Any additional relevant information is available from corresponding authors upon request. Previously published datasets used in this study are available at NCBI Gene Expression Omnibus: GSE148391 [72] and GSE34743 [73].

## Abbreviations

CPM(s): Count(s) per million
CRISPR: Clustered Regularly Interspaced Short Palindromic Repeats
FDR: False discovery rate
LNA: Locked nucleic acid
miR: MicroRNA
mRNA: Messenger RNA
MZT: Maternal-to-zygotic transition
PCA: Principal component analysis
qRT-PCR: Quantitative real-time polymerase chain reaction
RNAs: Ribonucleic acids
s4-UTP: 4-Thiouridine triphosphate
SLAM-seq: Thiol-linked alkylation for the metabolic sequencing of RNA
SNP(s): Single-nucleotide polymorphism(s)
T>C: Conversion of thymidine to cytidine
3′UTR: Three Prime Untranslated Region of mRNAs
5′UTR: Five Prime Untranslated Region of mRNAs
V>N: Conversion of non-thymine nucleotide (i.e. guanine, cytosine, adenine) to other nucleotides
ZCS: Zygotic component score
ZGA: Zygotic genome activation
zCPMs: Zygotic CPMs

## References

[1] Vastenhouw NL, Cao WX, Lipshitz HD (2019). The maternal-to-zygotic transition revisited. Development.

[2] Tadros W, Lipshitz HD (2009). The maternal-to-zygotic transition: a play in two acts. Development.

[3] Kushawah G, Hernandez-Huertas L, Abugattas-Nunez Del Prado J, Martinez-Morales JR, DeVore ML, Hassan H (2020). CRISPR-Cas13d Induces Efficient mRNA Knockdown in Animal Embryos. Dev Cell.

[4] Lee MT, Bonneau AR, Takacs CM, Bazzini AA, DiVito KR, Fleming ES (2013). Nanog, Pou5f1 and SoxB1 activate zygotic gene expression during the maternal-to-zygotic transition. Nature.

[5] Gagnon JA, Obbad K, Schier AF (2018). The primary role of zebrafish nanog is in extra-embryonic tissue. Development.

[6] Harvey SA, Sealy I, Kettleborough R, Fenyes F, White R, Stemple D (2013). Identification of the zebrafish maternal and paternal transcriptomes. Development.

[7] Chan SH, Tang Y, Miao L, Darwich-Codore H, Vejnar CE, Beaudoin JD (2019). Brd4 and P300 Confer Transcriptional Competency during Zygotic Genome Activation. Dev Cell.

[8] Laue K, Rajshekar S, Courtney AJ, Lewis ZA, Goll MG (2019). The maternal to zygotic transition regulates genome-wide heterochromatin establishment in the zebrafish embryo. Nat Commun.

[9] Heyn P, Kircher M, Dahl A, Kelso J, Tomancak P, Kalinka AT (2014). The earliest transcribed zygotic genes are short, newly evolved, and different across species. Cell Rep.

[10] Riemondy K, Henriksen JC, Rissland OS (2023). Intron dynamics reveal principles of gene regulation during the maternal-to-zygotic transition. RNA.

[11] Nudelman G, Frasca A, Kent B, Sadler KC, Sealfon SC, Walsh MJ (2018). High resolution annotation of zebrafish transcriptome using long-read sequencing. Genome Res.

[12] Liu Z, Wang W, Li X, Zhao X, Zhao H, Yang W (2022). Temporal dynamic analysis of alternative splicing during embryonic development in zebrafish. Front Cell Dev Biol.

[13] Ulitsky I, Shkumatava A, Jan CH, Subtelny AO, Koppstein D, Bell GW (2012). Extensive alternative polyadenylation during zebrafish development. Genome Res.

[14] Aanes H, Ostrup O, Andersen IS, Moen LF, Mathavan S, Collas P (2013). Differential transcript isoform usage pre- and post-zygotic genome activation in zebrafish. BMC Genomics.

[15] Haberle V, Li N, Hadzhiev Y, Plessy C, Previti C, Nepal C (2014). Two independent transcription initiation codes overlap on vertebrate core promoters. Nature.

[16] Nepal C, Hadzhiev Y, Previti C, Haberle V, Li N, Takahashi H (2013). Dynamic regulation of the transcription initiation landscape at single nucleotide resolution during vertebrate embryogenesis. Genome Res.

[17] Jukam D, Shariati SAM, Skotheim JM (2017). Zygotic Genome Activation in Vertebrates. Dev Cell.

[18] Drexler HL, Choquet K, Churchman LS (2020). Splicing Kinetics and Coordination Revealed by Direct Nascent RNA Sequencing through Nanopores. Mol Cell.

[19] Reimer KA, Mimoso CA, Adelman K, Neugebauer KM (2021). Co-transcriptional splicing regulates 3' end cleavage during mammalian erythropoiesis. Mol Cell.

[20] Herzog VA, Reichholf B, Neumann T, Rescheneder P, Bhat P, Burkard TR (2017). Thiol-linked alkylation of RNA to assess expression dynamics. Nat Methods.

[21] Bhat P, Cabrera-Quio LE, Herzog VA, Fasching N, Pauli A, Ameres SL (2023). SLAMseq resolves the kinetics of maternal and zygotic gene expression during early zebrafish embryogenesis. Cell Rep.

[22] Despic V, Neugebauer KM (2018). RNA tales - how embryos read and discard messages from mom. J Cell Sci.

[23] Despic V, Dejung M, Gu M, Krishnan J, Zhang J, Herzel L (2017). Dynamic RNA-protein interactions underlie the zebrafish maternal-to-zygotic transition. Genome Res.

[24] Shi B, Zhang J, Heng J, Gong J, Zhang T, Li P (2020). RNA structural dynamics regulate early embryogenesis through controlling transcriptome fate and function. Genome Biol.

[25] Chang H, Yeo J, Kim JG, Kim H, Lim J, Lee M (2018). Terminal Uridylyltransferases Execute Programmed Clearance of Maternal Transcriptome in Vertebrate Embryos. Mol Cell.

[26] Yang Y, Wang L, Han X, Yang WL, Zhang M, Ma HL (2019). RNA 5-Methylcytosine Facilitates the Maternal-to-Zygotic Transition by Preventing Maternal mRNA Decay. Mol Cell.

[27] Buchumenski I, Holler K, Appelbaum L, Eisenberg E, Junker JP, Levanon EY (2021). Systematic identification of A-to-I RNA editing in zebrafish development and adult organs. Nucleic Acids Res.

[28] Bazzini AA, Del Viso F, Moreno-Mateos MA, Johnstone TG, Vejnar CE, Qin Y (2016). Codon identity regulates mRNA stability and translation efficiency during the maternal-to-zygotic transition. EMBO J.

[29] Medina-Munoz SG, Kushawah G, Castellano LA, Diez M, DeVore ML, Salazar MJB (2021). Crosstalk between codon optimality and cis-regulatory elements dictates mRNA stability. Genome Biol.

[30] Mishima Y, Tomari Y (2016). Codon usage and 3' UTR length determine maternal mRNA stability in zebrafish. Mol Cell.

[31] Wu Q, Bazzini AA (2023). Translation and mRNA stability control. Annu Rev Biochem.

[32] Giraldez AJ, Mishima Y, Rihel J, Grocock RJ, Van Dongen S, Inoue K (2006). Zebrafish MiR-430 promotes deadenylation and clearance of maternal mRNAs. Science.

[33] Hadzhiev Y, Wheatley L, Cooper L, Ansaloni F, Whalley C, Chen Z (2023). The miR-430 locus with extreme promoter density forms a transcription body during the minor wave of zygotic genome activation. Dev Cell.

[34] Choi WY, Giraldez AJ, Schier AF (2007). Target protectors reveal dampening and balancing of Nodal agonist and antagonist by miR-430. Science.

[35] Brown W, Bardhan A, Darrah K, Tsang M, Deiters A (2022). Optical control of MicroRNA function in zebrafish embryos. J Am Chem Soc.

[36] Takacs CM, Giraldez AJ (2016). miR-430 regulates oriented cell division during neural tube development in zebrafish. Dev Biol.

[37] Liu Y, Zhu Z, Ho IHT, Shi Y, Li J, Wang X (2020). Genetic deletion of miR-430 disrupts maternal-zygotic transition and embryonic body plan. Front Genet.

[38] Zhao BS, Wang X, Beadell AV, Lu Z, Shi H, Kuuspalu A (2017). m(6)A-dependent maternal mRNA clearance facilitates zebrafish maternal-to-zygotic transition. Nature.

[39] Muhar M, Ebert A, Neumann T, Umkehrer C, Jude J, Wieshofer C (2018). SLAM-seq defines direct gene-regulatory functions of the BRD4-MYC axis. Science.

[40] Bazzini AA, Lee MT, Giraldez AJ (2012). Ribosome profiling shows that miR-430 reduces translation before causing mRNA decay in zebrafish. Science.

[41] Vejnar CE, Abdel Messih M, Takacs CM, Yartseva V, Oikonomou P, Christiano R (2019). Genome wide analysis of 3' UTR sequence elements and proteins regulating mRNA stability during maternal-to-zygotic transition in zebrafish. Genome Res.

[42] Solnica-Krezel L (2020). Maternal contributions to gastrulation in zebrafish. Curr Top Dev Biol..

[43] Schulz KN, Harrison MM (2019). Mechanisms regulating zygotic genome activation. Nat Rev Genet.

[44] Lee MT, Bonneau AR, Giraldez AJ (2014). Zygotic genome activation during the maternal-to-zygotic transition. Annu Rev Cell Dev Biol.

[45] Eckersley-Maslin MA, Alda-Catalinas C, Reik W (2018). Dynamics of the epigenetic landscape during the maternal-to-zygotic transition. Nat Rev Mol Cell Biol.

[46] Kaaij LJT, van der Weide RH, Ketting RF, de Wit E (2018). Systemic Loss and Gain of Chromatin Architecture throughout Zebrafish Development. Cell Rep.

[47] Zhang B, Wu X, Zhang W, Shen W, Sun Q, Liu K (2018). Widespread Enhancer Dememorization and Promoter Priming during Parental-to-Zygotic Transition. Mol Cell.

[48] Palfy M, Schulze G, Valen E, Vastenhouw NL (2020). Chromatin accessibility established by Pou5f3, Sox19b and Nanog primes genes for activity during zebrafish genome activation. PLoS Genet.

[49] Potok ME, Nix DA, Parnell TJ, Cairns BR (2013). Reprogramming the maternal zebrafish genome after fertilization to match the paternal methylation pattern. Cell.

[50] Jiang L, Zhang J, Wang JJ, Wang L, Zhang L, Li G (2013). Sperm, but not oocyte, DNA methylome is inherited by zebrafish early embryos. Cell.

[51] Wike CL, Guo Y, Tan M, Nakamura R, Shaw DK, Diaz N (2021). Chromatin architecture transitions from zebrafish sperm through early embryogenesis. Genome Res.

[52] Hickey GJ, Wike CL, Nie X, Guo Y, Tan M, Murphy PJ (2022). Establishment of developmental gene silencing by ordered polycomb complex recruitment in early zebrafish embryos. Elife.

[53] Liu G, Wang W, Hu S, Wang X, Zhang Y (2018). Inherited DNA methylation primes the establishment of accessible chromatin during genome activation. Genome Res.

[54] Miao L, Tang Y, Bonneau AR, Chan SH, Kojima ML, Pownall ME (2022). The landscape of pioneer factor activity reveals the mechanisms of chromatin reprogramming and genome activation. Mol Cell.

[55] Joseph SR, Palfy M, Hilbert L, Kumar M, Karschau J, Zaburdaev V (2017). Competition between histone and transcription factor binding regulates the onset of transcription in zebrafish embryos. Elife.

[56] Veil M, Yampolsky LY, Gruning B, Onichtchouk D (2019). Pou5f3, SoxB1, and Nanog remodel chromatin on high nucleosome affinity regions at zygotic genome activation. Genome Res.

[57] Hernandez-Huertas L, Kushawah G, Diaz-Moscoso A, Tomas-Gallardo L, Moreno-Sanchez I, da Silva Pescador G (2022). Optimized CRISPR-RfxCas13d system for RNA targeting in zebrafish embryos. STAR Protoc.

[58] Serobyan V, Kontarakis Z, El-Brolosy MA, Welker JM, Tolstenkov O, Saadeldein AM (2020). Transcriptional adaptation in Caenorhabditis elegans. Elife.

[59] Ma Z, Zhu P, Shi H, Guo L, Zhang Q, Chen Y (2019). PTC-bearing mRNA elicits a genetic compensation response via Upf3a and COMPASS components. Nature.

[60] El-Brolosy MA, Kontarakis Z, Rossi A, Kuenne C, Gunther S, Fukuda N (2019). Genetic compensation triggered by mutant mRNA degradation. Nature.

[61] Lim LP, Lau NC, Garrett-Engele P, Grimson A, Schelter JM, Castle J (2005). Microarray analysis shows that some microRNAs downregulate large numbers of target mRNAs. Nature.

[62] Rhoades MW, Reinhart BJ, Lim LP, Burge CB, Bartel B, Bartel DP (2002). Prediction of plant microRNA targets. Cell.

[63] Larson ED, Marsh AJ, Harrison MM (2021). Pioneering the developmental frontier. Mol Cell.

[64] Gao M, Veil M, Rosenblatt M, Riesle AJ, Gebhard A, Hass H (2022). Pluripotency factors determine gene expression repertoire at zygotic genome activation. Nat Commun.

[65] Holler K, Neuschulz A, Drewe-Boss P, Mintcheva J, Spanjaard B, Arsie R (2021). Spatio-temporal mRNA tracking in the early zebrafish embryo. Nat Commun.

[66] Kimmel CB, Ballard WW, Kimmel SR, Ullmann B, Schilling TF (1995). Stages of embryonic development of the zebrafish. Dev Dyn.

[67] Livak KJ, Schmittgen TD (2001). Analysis of relative gene expression data using real-time quantitative PCR and the 2(-Delta Delta C(T)) Method. Methods.

[68] Krueger F, James F, Ewels P, Afyounian E, Weinstein M. FelixKrueger/TrimGalore: v0.6.10 - add default decompression path (0.6.10). Zenodo2023.

[69] Robinson MD, McCarthy DJ, Smyth GK (2010). edgeR: a Bioconductor package for differential expression analysis of digital gene expression data. Bioinformatics.

[70] Yu G, Wang LG, Han Y, He QY (2012). clusterProfiler: an R package for comparing biological themes among gene clusters. OMICS.

[71] Baia Amaral D, Egidy R, Perera A, Bazzini A. Slam-seq reveals that miR-430 regulates zygotic mRNA during zebrafish embryogenesis. GSE247935. Gene Expression Omnibus. https://www.ncbi.nlm.nih.gov/geo/query/acc.cgi?acc=GSE247935. (2024). 10.1186/s13059-024-03197-8 PMC10949700 38504288

[72] Bazzini A, Medina S. Early zebrafish development time course transcriptome. GSE148391. Gene Expression Omnibus. https://www.ncbi.nlm.nih.gov/geo/query/acc.cgi?acc= GSE148391 (2020).

[73] Bazzini A, Lee M, Giraldez A. Ribosome profiling of early zebrafish embryos -- miRNA-mediated regulation during embryogenesis causes translational repression before mRNA decay. GSE34743. Gene Expression Omnibus. https://www.ncbi.nlm.nih.gov/geo/query/acc.cgi?acc=GSE34743. (2012).

