# Supplementary material for: miR-430 regulates zygotic mRNA during zebrafish embryogenesis
Source: Genome Biol. 2024 Mar 19;25:74. doi: 10.1186/s13059-024-03197-8 (PMC10949700; doi:10.1186/s13059-024-03197-8)
Supplement: Supplementary file 1 — Additional file 1: Fig. S1. Zebrafish embryos development after s4-UTP injections, SLAM-seq/Quant-seq transcriptomic comparisons of wild-type, α-Amanitin injected embryos to regular Poly-A enriched RNA-seq. SLAM-seq ‘T’ to ‘C’ rates reproducibility and correlation between labeled reads percentage and UTR length, ‘T’ content or RNA level. [file 13059_2024_3197_MOESM1_ESM.pdf]

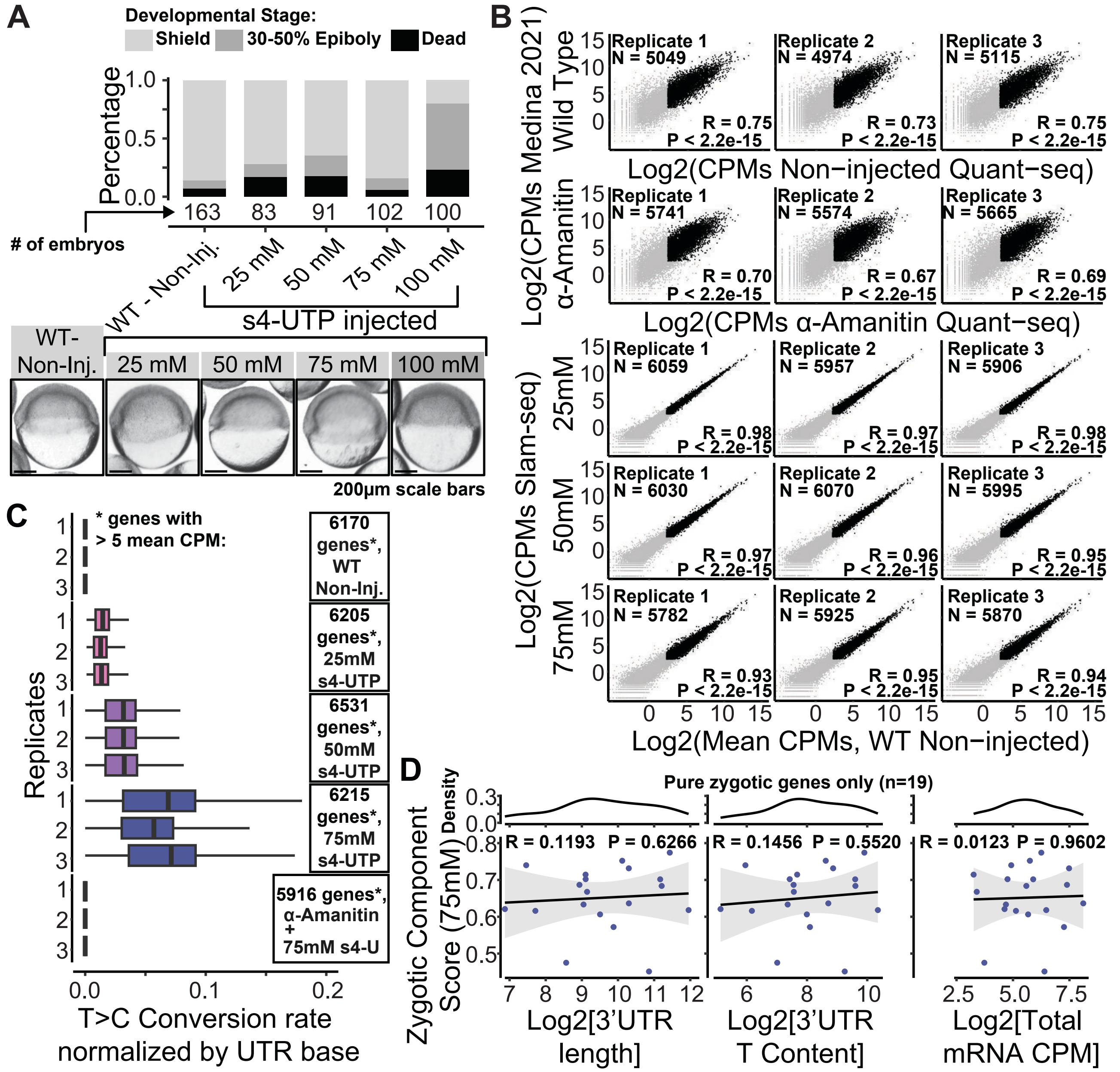

Fig S1. s4-UTP injections and SLAM-seq show no significant developmental delay or global changes in gene expression. (A) Stacked bar plots showing the percentage of embryos in each developmental stage at ~6 hours post-injection from each group (including number of fertilized eggs). Insets include representative pictures of the most frequent developmental stage observed in each group (scale bars = 200µm). (B) Scatter plots showing pairwise comparisons of SLAM-seq and Poly-A RNA-seq (Medina et al., 2021) counts per million (CPMs). Means or individual replicates indicated in the figure. Spearman correlation coefficient and p-values from genes expressed (>5 CPMs, black dots) in both conditions being compared are indicated. N = number of expressed genes. (C) Boxplot showing T>C conversion rate normalized by 3'UTR base of all expressed genes in each replicate per condition (n, number of genes >5 mean counts per million, CPMs). (D) Scatterplots overlayed linear regression line (95% confidence intervals), showing correlation between mean zygotic component score of 19 pure zygotic genes from SLAM-seq (75mM s4-UTP injections) and one of: 3'UTR length, 3'UTR ‘T’ content, mean total mRNA CPMs (counts per million). Spearman correlation coefficient and p-values showed. Density plot from each comparison is showed on top of each graph, highlighting the distribution of features among the genes analyzed.
