# Supplementary material for: miR-430 regulates zygotic mRNA during zebrafish embryogenesis
Source: Genome Biol. 2024 Mar 19;25:74. doi: 10.1186/s13059-024-03197-8 (PMC10949700; doi:10.1186/s13059-024-03197-8)
Supplement: Supplementary file 3 — Additional file 3: Fig. S2. SLAM-seq replicate and time course grouping analysis, detection of increasing labeled reads percentage, false positive labeling events and pipeline for gene class identification (Pure Maternal, Maternal-zygotic, Pure Zygotic). [file 13059_2024_3197_MOESM3_ESM.pdf]

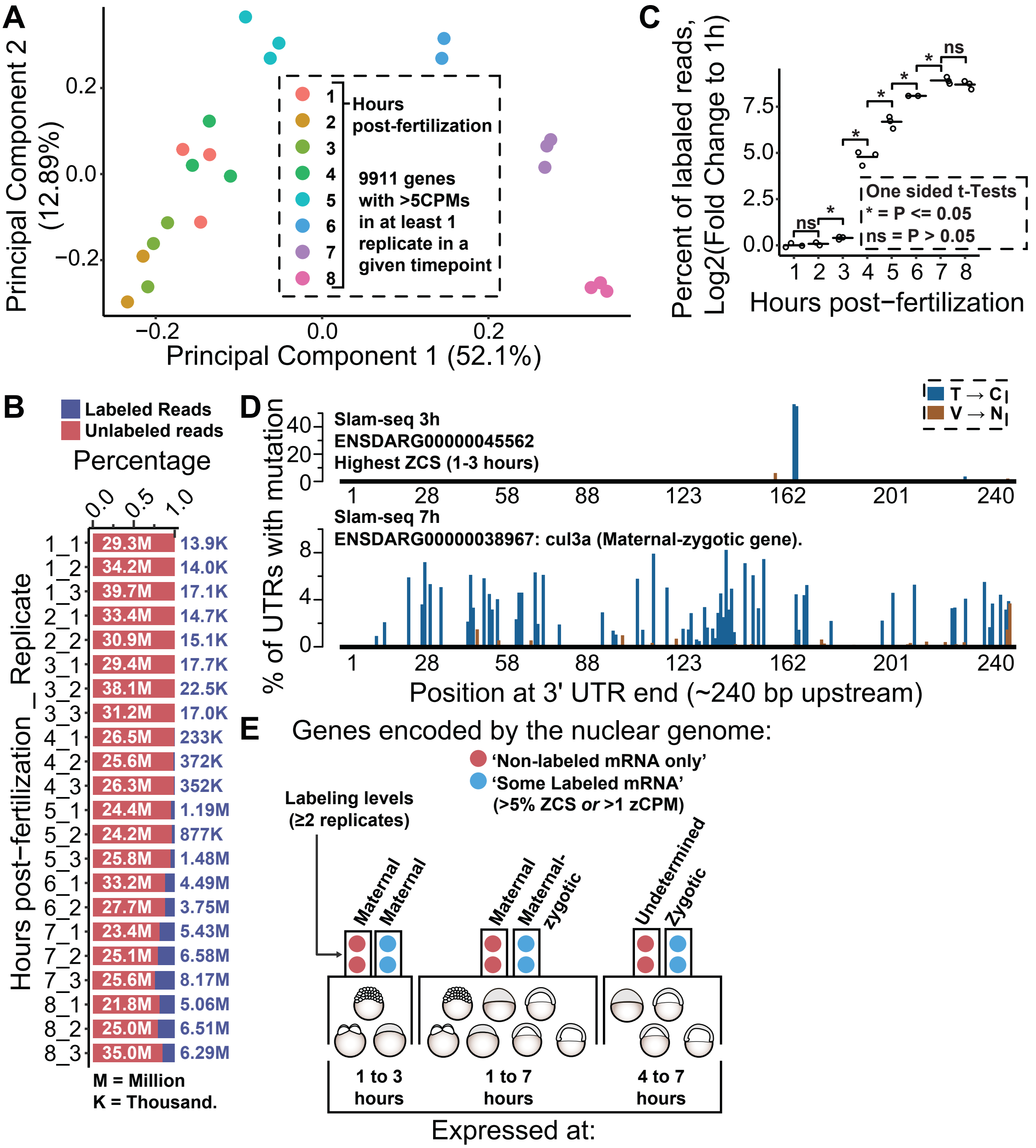

Fig S2. SLAM-seq highlights significant onset of zygotic genome transcription at 3-4 hours. (A) Scatterplot showing results from Principal Component analysis for all genes expressed in at least one sample (> 5 counts per million) throughout the 8 hours of zebrafish development. (B) Scatterplot and mean bars for fold change of percent of labeling of all timepoints compared to 1h (top). Stacked bar plots showing percent of labeled (blue) and unlabeled (red) reads in each replicate for each timepoint. The absolute number of mapped reads for each category are indicated. (C) Histograms showing relative frequency (percent of 3'UTRs with a given mutation) of T>C and V>N mutations in a representative example along the last 250 base pairs of the transcript expressed 1-3 hours (top) and 1-7h (bottom). (D) Schematic representation of time-course-wide gene classification using SLAM-seq data, in short: expressed genes (>5 CPMs, counts per million) with significant labeling (>5% zygotic component score or > 1 zygotic CPM) in at least two replicates are considered zygotically expressed; if genes are expressed only after 3 hours and have reproducible labeling, they are termed pure zygotic, if they lack reproducible labeling, they are deemed undetermined. All genes expressed at 1-3 hours without significant reproducible labeling after are deemed pure maternal, and the ones with reproducible labeling after 3 hours, maternal-zygotic.
