# Supplementary material for: miR-430 regulates zygotic mRNA during zebrafish embryogenesis
Source: Genome Biol. 2024 Mar 19;25:74. doi: 10.1186/s13059-024-03197-8 (PMC10949700; doi:10.1186/s13059-024-03197-8)
Supplement: Supplementary file 4 — Additional file 4: Fig. S3. Results from Gene Ontology enrichment analysis for hourly grouped transcriptional activation events. [file 13059_2024_3197_MOESM4_ESM.pdf]

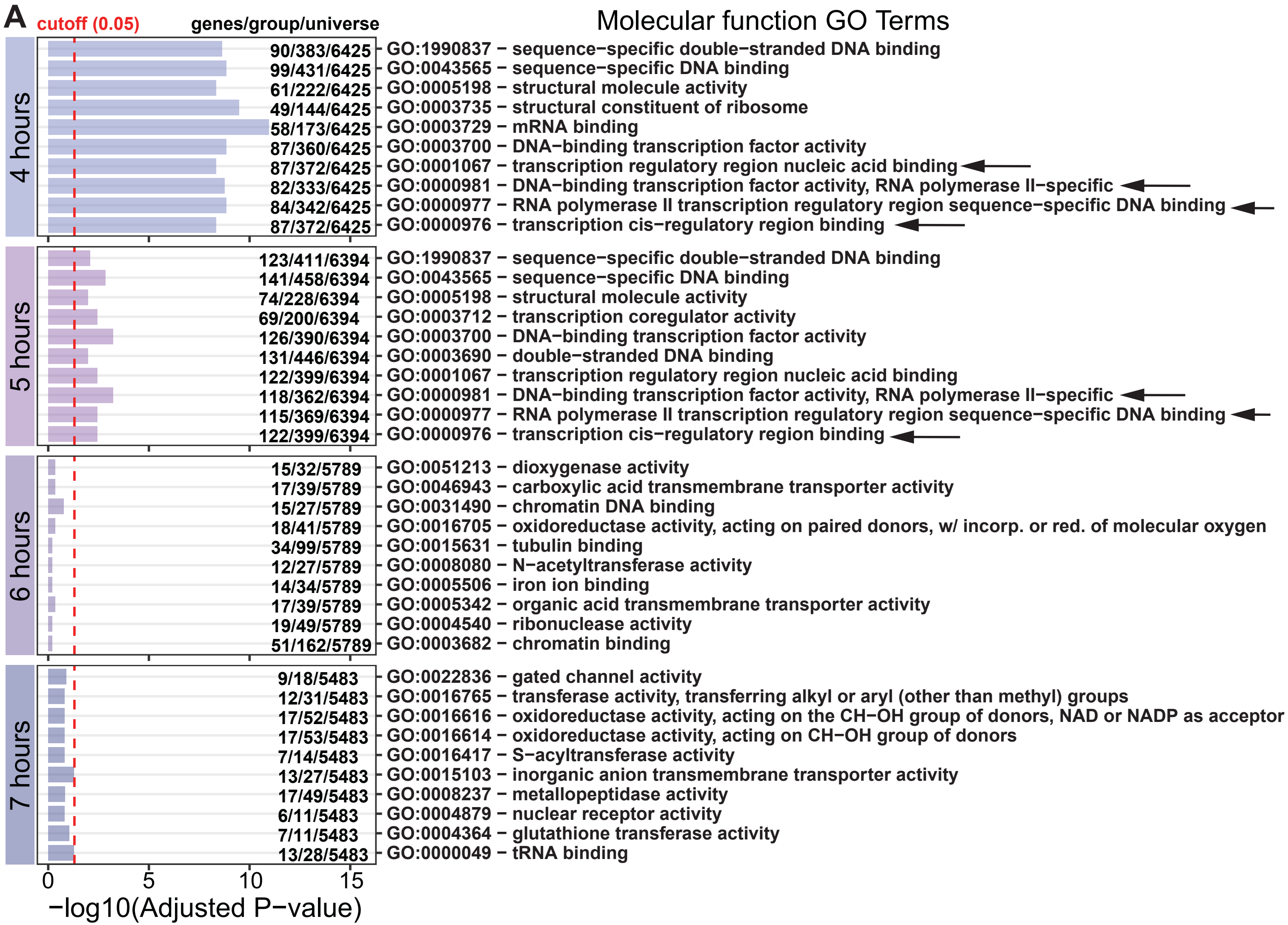

Fig S3. SLAM-seq hourly grouped transcriptional activation events show gene enrichment terms related to RNA life cycle and transcription. (A) Side bar plots showing adjusted p-values and GO terms for molecular processes enriched in each hourly grouped transcriptional activation events (Figure 3 B). Number of found genes per enriched group, the total number of genes annotated in that group and the universe of genes (expressed genes at that given timepoint) are shown.
