# Supplementary material for: miR-430 regulates zygotic mRNA during zebrafish embryogenesis
Source: Genome Biol. 2024 Mar 19;25:74. doi: 10.1186/s13059-024-03197-8 (PMC10949700; doi:10.1186/s13059-024-03197-8)
Supplement: Supplementary file 5 — Additional file 5: Fig. S4. Titration of s4-UTP and LNAs injection, assessment of miR-430 targets de-repression at shield stage and phenotype at one day post-fertilization. [file 13059_2024_3197_MOESM5_ESM.pdf]

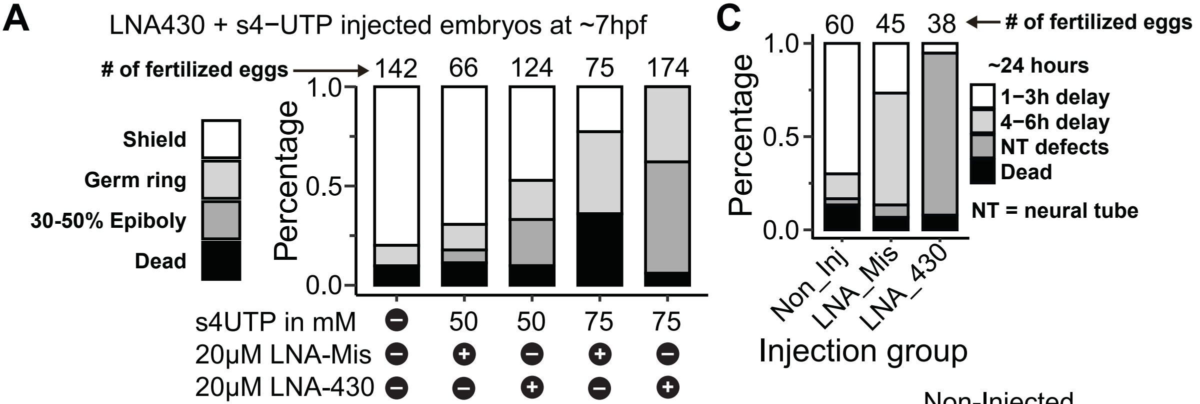

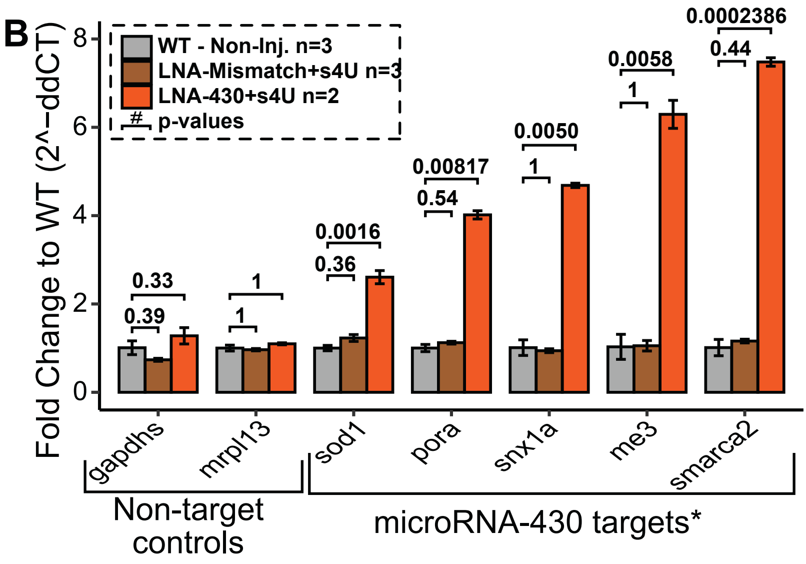

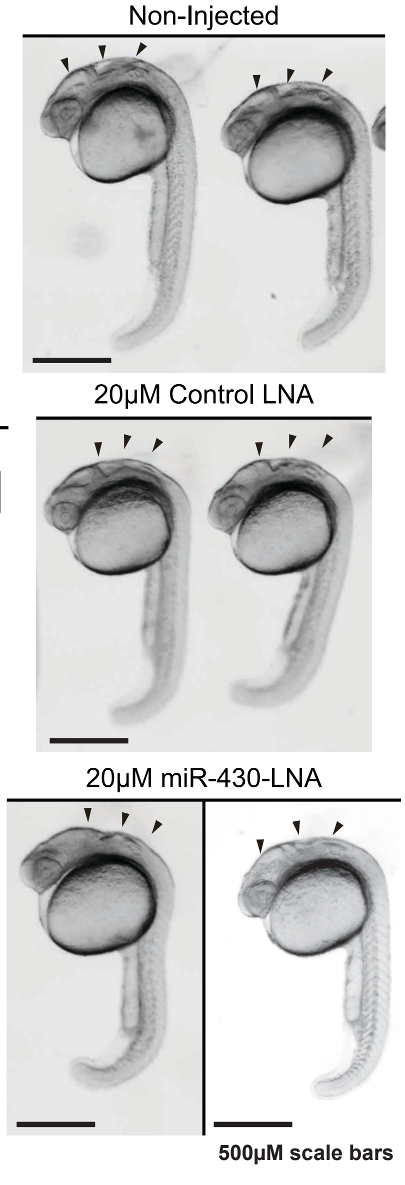

Fig S4. SLAM-seq can be used along with microRNA-430 loss-of-function to study microRNA function during zebrafish development. (A, C) Stacked bar plots showing the percentage of embryos in each developmental stage at ~6 (A) and ~24 (C) hours post-injection from each group (including number of fertilized eggs), including representative pictures observed in each group (C, scale bars = 500μm). (B) Bar plot showing Fold changes to WT-non-injected embryos of embryos injected with 50mM s4UTP and either Control LNA or miR-430-LNA. Non-target controls and previously reported miR-430 targets are shown, values represent mean +/- standard deviation of each gene for each group. P-values from t-tests are shown for each pairwise comparison, along with number of biological replicates per group.
