# Supplementary material for: miR-430 regulates zygotic mRNA during zebrafish embryogenesis
Source: Genome Biol. 2024 Mar 19;25:74. doi: 10.1186/s13059-024-03197-8 (PMC10949700; doi:10.1186/s13059-024-03197-8)
Supplement: Supplementary file 7 — Additional file 7. Peer review history. [file 13059_2024_3197_MOESM7_ESM.docx]

**Review history**

**First round of review**

**Reviewer 1**

In this manuscript, Baia Amaral et al., report their new findings on microRNA-mediated clearance of mRNA during the maternal-to-zygotic transition. The authors use the metabolic labeling of mRNA (SLAM-Seq) to discern between maternally-provided and newly transcribed mRNAs during early zebrafish embryogenesis. Previous works have attempted to dissect the maternal mRNA clearance dynamics using bulk RNA sequencing and have successfully identified miR-430 and several RNA-binding proteins as main players of this process. However, bulk RNA sequencing relies on large changes of mRNA levels between maternal and zygotic stages to identify actively degraded transcripts. Therefore, this approach is blind to the large proportion of genes that remain at stable levels between the two developmental stages. Metabolic labeling of mRNA using SLAM-Seq allows to resolve the problem of distinguishing maternal and zygotic mRNAs with the same sequence.

Despite the previous work of Bhat et al. applied SLAM-Seq on zebrafish embryos, which the authors rightfully acknowledge throughout the manuscript, the current manuscript reports novel insights into maternal-to-zygotic transcription. The current work specifically focuses on the miRNA-mediated clearance of mRNA while Bhat et al. focused on determining which genes were transcribed during early embryogenesis. In the current manuscript, Baia Amaral et al. uncover that even de novo synthetized mRNAs undergo some degree of degradation instructed by miR-430. The microRNA-mediated regulation needs to be factored in when calculating transcriptional rates just based on RNA levels. This manuscript also demonstrates that microRNAs can have an all or nothing response for certain mRNAs (maternal mRNAs) or act more as a fine tuning of gene expression.

To achieve these results, the authors thoroughly validate the robustness of their metabolic labeling by blocking transcription, in which case the modified nucleotide 4SU cannot be added to the mRNA. In addition, the authors use tiny LNAs against miR-430 to uncouple microRNA-mediated mRNA decay and experimentally validate real targets of miR-430.

Overall, this manuscript presents a new point of view of the relevant topic of post-transcriptional regulation during maternal-to-zygotic transition. The work is technically sound, the data is presented clearly (albeit the writing should be enhanced) and the results about mRNA degradation are novel an complement recent previous work focused on de novo transcription. Therefore, this manuscript is of interest for the broad audience of Genome Biology and is suitable for publication once the authors address the following minor concerns:

1 - References for Figure 2B and C in the text are mixed. The authors should cite the correct figures in the text.

2 - The labels on Figure 4 does not correspond to the annotations in the figure legend or in the main text.

3 - Some sentences should be rewritten to facilitate the flow of the reading. Sentences such "Yet its effects on the as well as the decay activity of pure zygotic genes and maternal or zygotic fraction of maternal-zygotic genes have been obscured by the lack of quantitatively disentangling them from each other" (lines 84-86) are confuse and need rewriting for clarity.

**Reviewer 2**

The manuscript by Amaral et al., describes a metabolic labelling approach in characterising the de novo transcriptome in early zebrafish embryos. The authors describe transcriptional waves, suggest that most genes of the early embryo are expressed by both maternal and zygotic contributions and they demonstrate by SLAM-seq combined with miR-430 interference that many zygotically active genes including those that are also deposited maternally, are targeted by miR-430, key regulator of maternal mRNA degradation in zebrafish, for modulating their overall expression levels.

Zygotic genome activation and the maternal to zygotic transition has been subjected to many genomics studies in zebrafish and other animal models yet fundamental questions remain about the role and mechanisms of waves of degradation and activation of transcriptomes during this period. SLAM-seq is a powerful method to detect nascent RNAs. This technique is particularly useful during the maternal to zygotic transition, which is characterised by major turnover of transcripts deposited by the mother into the egg and concomitant gradual but pervasive activation of the zygotic genome. Since these processes act in parallel, disentangling the maternal and zygotic components of genes expressed throughout early development has been a challenge. This study offers detailed (hourly) description of zygotic transcription and offers refinements of how and which genes get activated during blastula/early gastrula stages.
However, it is not apparent to this reviewer how novel the findings presented in this study are. It is arguable, whether the results go beyond confirmation or offer substantial advance on our understanding of the dynamics of transcriptional and post-transcriptional regulation of genome activation:
The authors claim that their study demonstrates that over half of the genes are maternal and zygotic. This has been shown before multiple times (also hinted at by the authors) and well established in the field (reviewed in e.g. PMID: 31189646). The most recent SLAM-seq study pointing this out is cited by the ms PMID: 24531765. The authors further argue that there is a lack of understanding of the contribution of maternal and zygotic components to maternal-zygotic gene activities in the lack of quantitative disentangling them. This is not precise, as the other SLAM-seq based study in Cell Reports illustrates and there also was separation of maternal and zygotic components of activities by promoter variation using CAGE-seq (PMID: 24531765).

The other main claim of the study concerns the role of miR-430 in posttranscriptional modulation of hundreds of zygotically transcribed genes. That miR-430 targets zygotic genes besides maternal transcripts has been demonstrated before (noted in the introduction). The original paper by Giraldez et al., PMID: 16484454 shows zygotic targets in Figure 4 and also states that 'most' miR-430 targets are expressed maternally, i.e. not all of them are maternal. In addition, PMID: 32849832 showed that MZ and zygotic genes are also upregulated in miR430 deletion mutants. Furthermore, miR-430 is known to be expressed beyond ZGA and has also been shown to affect development beyond ZGA (PMID: 36073798). While the ms by Amarel et al. offers direct and more robust analysis of miR-430 zygotic targets, however the suggestion that their results propose a new role for miR-430, needs refining. The scale of miR-430 regulation of zygotic genes is potentially interesting if the study would go beyond confirming this by loss of function of miR-430 and would offer more in depth of analysis that may hint at functional or regulatory relevance of this fine tuning in early development.

The third main claim of the manuscript is a proposal for an hourly wave of gene activation dynamic during ZGA. Such an hourly wave would only be possible to detect if the sampling was sufficiently frequent (several time points per hour) to distinguish true waves from potential effect of timing of sampling.

Taken together, in its current form the findings of the Amarales et al., manuscript are either confirmatory or somewhat preliminary which together dampens this reviewer's enthusiasm.


Minor points
1. The authors describe titration of the 4-SU labelling and indicate that using 75 mM embryos develop normally, whereas in 100 mM the embryos arrest at sphere. This is a very small difference in concentration. It is conceivable, that injection volume variation could lead to a proportion of embryos in the pools being affected by receiving toxic levels of the reagent. How did the authors confirm the viability of embryos?
2. Large body of literature of ZGA regulation is not reflected by the comparably small number of references cited in this ms.
3. There is need for checking the ms for typos and grammatical errors. Without comprehensive list, some examples are below:
SLAM is written with all caps
Line 207: 'miR-430 evidences' - this does not make clear sense.
Figure 2D caption: 'Non-labaled'.
Line 83 'clearer'.
Figure 3C: 'Cahange'.
'Replica' should be replaced with 'Replicate' in the Supp. Figures.
Font sizes and styles (use of bold) ought to be harmonised for easier reading and interpretation of the densely presented Figures.

**Authors’ response to reviewers**

Reviewer #1
In this manuscript, Baia Amaral et al., report their new findings on microRNA-mediated clearance of mRNA during the maternal-to-zygotic transition. The authors use the metabolic labeling of mRNA (SLAM-Seq) to discern between maternally-provided and newly transcribed mRNAs during early zebrafish embryogenesis. Previous works have attempted to dissect the maternal mRNA clearance dynamics using bulk RNA sequencing and have successfully identified miR-430 and several RNA-binding proteins as main players of this process. However, bulk RNA sequencing relies on large changes of mRNA levels between maternal and zygotic stages to identify actively degraded transcripts. Therefore, this approach is blind to the large proportion of genes that remain at stable levels between the two developmental stages. Metabolic labeling of mRNA using SLAM-Seq allows to resolve the problem of distinguishing maternal and zygotic mRNAs with the same sequence.

Despite the previous work of Bhat et al. applied SLAM-Seq on zebrafish embryos, which the authors rightfully acknowledge throughout the manuscript, the current manuscript reports novel insights into maternal-to-zygotic transcription. The current work specifically focuses on the miRNA-mediated clearance of mRNA while Bhat et al. focused on determining which genes were transcribed during early embryogenesis. In the current manuscript, Baia Amaral et al. uncover that even de novo synthetized mRNAs undergo some degree of degradation instructed by miR-430. The microRNA-mediated regulation needs to be factored in when calculating transcriptional rates just based on RNA levels. This manuscript also demonstrates that microRNAs can have an all or nothing response for certain mRNAs (maternal mRNAs) or act more as a fine tuning of gene expression.

To achieve these results, the authors thoroughly validate the robustness of their metabolic labeling by blocking transcription, in which case the modified nucleotide 4SU cannot be added to the mRNA. In addition, the authors use tiny LNAs against miR-430 to uncouple microRNA-mediated mRNA decay and experimentally validate real targets of miR-430.

Overall, this manuscript presents a new point of view of the relevant topic of post-transcriptional regulation during maternal-to-zygotic transition. The work is technically sound, the data is presented clearly (albeit the writing should be enhanced) and the results about mRNA degradation are novel an complement recent previous work focused on de novo transcription. Therefore, this manuscript is of interest for the broad audience of Genome Biology and is suitable for publication once the authors address the following minor concerns:
Authors’ Comments: We appreciate your kind comments and feedback and have incorporated them into the text.

#To Referee 1 comments:
“1 - References for Figure 2B and C in the text are mixed. The authors should cite the correct figures in the text.
Authors’ Comments: Figure references are now corrected.
2 - The labels on Figure 4 does not correspond to the annotations in the figure legend or in the main text.
Authors’ Comments: Figure legends and references are now corrected.
3 - Some sentences should be rewritten to facilitate the flow of the reading. Sentences such "Yet its effects on the as well as the decay activity of pure zygotic genes and maternal or zygotic fraction of maternal-zygotic genes have been obscured by the lack of quantitatively disentangling them from each other" (lines 84-86) are confuse and need rewriting for clarity.”
Authors’ Comments: These sentences have been re-written to follow reviewer’s suggestions.

Reviewer #2
The manuscript by Amaral et al., describes a metabolic labelling approach in characterising the de novo transcriptome in early zebrafish embryos. The authors describe transcriptional waves, suggest that most genes of the early embryo are expressed by both maternal and zygotic contributions and they demonstrate by SLAM-seq combined with miR-430 interference that many zygotically active genes including those that are also deposited maternally, are targeted by miR-430, key regulator of maternal mRNA degradation in zebrafish, for modulating their overall expression levels.

Zygotic genome activation and the maternal to zygotic transition has been subjected to many genomics studies in zebrafish and other animal models yet fundamental questions remain about the role and mechanisms of waves of degradation and activation of transcriptomes during this period. SLAM-seq is a powerful method to detect nascent RNAs. This technique is particularly useful during the maternal to zygotic transition, which is characterised by major turnover of transcripts deposited by the mother into the egg and concomitant gradual but pervasive activation of the zygotic genome. Since these processes act in parallel, disentangling the maternal and zygotic components of genes expressed throughout early development has been a challenge. This study offers detailed (hourly) description of zygotic transcription and offers refinements of how and which genes get activated during blastula/early gastrula stages.
However, it is not apparent to this reviewer how novel the findings presented in this study are. It is arguable, whether the results go beyond confirmation or offer substantial advance on our understanding of the dynamics of transcriptional and post-transcriptional regulation of genome activation:
Authors’ Comments: We appreciate your time in reviewing our manuscript and are committed to incorporating your constructive feedback. Please see below each of the responses.


#To Referee 2 comment #1:
“The authors claim that their study demonstrates that over half of the genes are maternal and zygotic. This has been shown before multiple times (also hinted at by the authors) and well established in the field (reviewed in e.g. PMID: 31189646). The most recent SLAM-seq study pointing this out is cited by the ms PMID: 24531765.”

Authors’ Comments: As highlighted by the reviewer, we recognize that the prevalence of maternal-zygotic genes has been extensively studied using various techniques, including the recent Slam-seq paper and this knowledge is well-established in the field. We did not intend to claim the discovery of the predominance of maternal and zygotic genes. In light of this, we have reworded the relevant sentences and included more references, including the addition of PMID: 31189646. However, we believe it is crucial to underscore that our Slam-seq also successfully identified a significant number of maternal and zygotic genes. We emphasize the reproducibility and resolution of Slam-seq, showcasing its promise for future investigations. Importantly, the conclusions drawn from Figures 1 and 2 are foundational for our subsequent analyses in Figures 3, 4, and 5. Recognizing the value in the reviewer's insights, we have adjusted our assertions in those respective sections.

#To Referee 2 comment #2:
“The authors further argue that there is a lack of understanding of the contribution of maternal and zygotic components to maternal-zygotic gene activities in the lack of quantitative disentangling them. This is not precise, as the other SLAM-seq based study in Cell Reports illustrates and there also was separation of maternal and zygotic components of activities by promoter variation using CAGE-seq (PMID: 24531765).”
Authors’ Comments: We genuinely recognize the reviewer's concerns and had no intention of overlooking prior contributions. In response, we have included a more exhaustive list of studies that have explored this topic, while also emphasizing the unique quantitative capabilities of SLAM-seq. As noted by the reviewer, the recently published Slam-seq study (PMID: 24531765) primarily centered on a select group of maternal-zygotic genes, largely analyzing their turnover rates, indicative of maternal mRNA decay. Concurrently, the CAGE-seq study (PMID: 24531765) delved into alterations in promoter usage for genes with distinguishable 5’UTRs/promoters. We deeply acknowledge the foundational role of these prior studies. Our revised claims now underscore the distinctiveness and innovation of our analysis, ensuring that our contributions to the field are evident.

#To Referee 2 comment #3:
“The other main claim of the study concerns the role of miR-430 in posttranscriptional modulation of hundreds of zygotically transcribed genes. That miR-430 targets zygotic genes besides maternal transcripts has been demonstrated before (noted in the introduction). The original paper by Giraldez et al., PMID: 16484454 shows zygotic targets in Figure 4 and also states that 'most' miR-430 targets are expressed maternally, i.e. not all of them are maternal. In addition, PMID: 32849832 showed that MZ and zygotic genes are also upregulated in miR430 deletion mutants. Furthermore, miR-430 is known to be expressed beyond ZGA and has also been shown to affect development beyond ZGA (PMID: 36073798). While the ms by Amarel et al. offers direct and more robust analysis of miR-430 zygotic targets, however the suggestion that their results propose a new role for miR-430, needs refining. The scale of miR-430 regulation of zygotic genes is potentially interesting if the study would go beyond confirming this by loss of function of miR-430 and would offer more in depth of analysis that may hint at functional or regulatory relevance of this fine tuning in early development.”
Authors’ Comments: As the reviewer rightly pointed out, prior studies, notably those by Giraldez et al. (PMID: 16484454) and PMID: 32849832, have indicated that certain zygotically expressed genes with miR-430 target sites exhibit upregulation in the absence of miR-430 function. Our Slam-seq experiments represent the first systematic approach that conclusively shows that miR-430 regulates zygotic mRNAs. Furthermore, our work stands as the first genome-wide analysis offering concrete direct proof that miR-430 also modulates the zygotic fraction of maternal-zygotic genes during the zebrafish MZT. It is essential to highlight that these antecedent studies did not provide direct evidence classifying these genes into distinct categories, such as Pure Maternal or Maternal-zygotic. Additionally, they did not demonstrate miR-430 influences the zygotic component of the Maternal-zygotic genes. Nonetheless, given their and others' discussions on the role of microRNAs in modulating mRNA levels of genes in active transcription (PMID: 15685193 and PMID: 12202040), we have now incorporated these references into our discussion section.
Next, and more specifically about PMID: 32849832, as well-spotted by the reviewer, others have looked at MZT Pure Zygotic miR-430 targets in the absence of miR-430, yet their analysis and interpretation arrive at a different conclusion than ours, as in lines: “We grouped the miR-430-regulated genes into three categories: (i) maternally provided transcripts that are rapidly degraded after ZGA; (ii) the steadily expressed genes during early development; and (iii) the up-regulated genes after ZGA (Figure 5A). Compared to the control data set (genes not significantly changed in the miR-430–/– mutants), the up-regulated genes in the miR-430–/– mutants were significantly enriched in category (i) (p = 3.0147E-41, Chi-squared test) but depleted in category (iii) (p = 2.6E-17, Chi-squared test) (Figures 5B,C), supporting that miR-430 is required for the clearance of maternally provided transcripts. Conversely, the down-regulated genes in the miR-430–/– mutants were significantly enriched in the category (iii) (p = 7.1E-18, Chi-squared test) but depleted in category (i) (p = 3.8E-28, Chi-squared test) (Figure 5D), demonstrating that the zygotic genome expressed genes were not fully activated in the miR-430–/– mutants”. They put emphasis on the role of miR-430 as maternal mRNA decay factor and concluded their miR-430 loss of function causes ZGA impairment (PMID: 32849832, figure 5 and discussion). Considering all these factors, we would like to clarify that our intent is not to critique their outstanding work, but rather to emphasize that the primary focus of our analysis differs from theirs. While they focus on maternal mRNA decay, our approach allows for the differentiation of the zygotic component, which is our focus. To make sure those differences are clear we have incorporated their conclusions into our discussion section.
Lastly, to the reviewer’s last point we recognized that the impact of miR-430 beyond these stages has been previously established (PMID: 36073798). We emphasize that our study specifically examines the function of miR-430 during the Maternal-to-Zygotic Transition (MZT) stages of early development. However, we have made these adjustments to our claims as we agree with the reviewer's observations and want to underscore the novelty of our study focused on miR-430 control over pure zygotic genes and zygotic fraction of maternal-zygotic genes during this time. We believe that these refinements will enhance the precision and clarity of our findings regarding the fine-tuning of zygotic mRNA expression during this critical developmental period.

#To Referee 2 comment #4:
“The third main claim of the manuscript is a proposal for an hourly wave of gene activation dynamic during ZGA. Such an hourly wave would only be possible to detect if the sampling was sufficiently frequent (several time points per hour) to distinguish true waves from potential effect of timing of sampling.”
Authors’ Comments: We are grateful for the reviewer's insights regarding the hourly wave of gene activation dynamics during ZGA. We have adjusted the manuscript, introducing qualifiers like 'to the extent of our sampling' to highlight the constraints related to our sampling frequency. Furthermore, we have included a discussion in the manuscript to explicitly address this limitation. We believe that these additions will provide a clearer perspective on the extent of our findings in relation to the sampling frequency.

#To Referee 2 comment #5:
“Taken together, in its current form the findings of the Amarales et al., manuscript are either confirmatory or somewhat preliminary which together dampens this reviewer's enthusiasm.”
Authors’ Comments: We genuinely appreciate the reviewer's feedback and understand their perspective. We have made several changes to the manuscript to enhance its value and novelty, addressing the concerns raised and committing ourselves to delivering a higher-quality contribution to the field by doing so. We would like to provide bullet points about the major contributions of our work:
• This is the second Slam-seq done in Zebrafish and the first Slam-seq performed in vivo with a chemical treatment (Alpha-amanitin) showing the specificity in capturing newly synthesized transcripts in vivo.
• This is also the first Slam-seq in vivo after manipulating regulatory gene function (LNA injections affecting miR-430 regulatory function). And represent the first systematic approach that conclusively shows that miR-430 regulates zygotic mRNAs. Moreover, this is the first genome-wide analysis providing direct evidence that miR-430 also modulates the zygotic fraction of maternal-zygotic genes during the zebrafish MZT.
• Additionally, we discuss how these experiments were important to interrogate whether the decay of miR-430 targets could trigger transcriptional adaptation (aka genetic compensation) response as previously suggested in other systems (PMID: 31951195).
• This also shows that Zebrafish-Slam-seq can be performed in conjunction with gene function manipulation. We evision that our work will be the entry point to start performing Slam-Seq in zebrafish embryos after knocking-down maternal genes using Cas13d-system, specifically factors related to ZGA.
• We are the first to specifically show timely genome activation on an unprecedent resolution genome wide including the maternal-zygotic gene group, which represents the largest group.
• We are the first to show long non-coding RNA gene classes as pure maternal or zygotic or maternal-zygotic using Slam-seq.
• Lastly, as discussed in our manuscript, newly labeled transcript changes across conditions during Slam-seq in cell culture assays has been interpreted as a proxy to transcription. However, our miR-430 experiments show a potential underestimation of transcription because Slam-seq is representing de novo mature mRNA levels. Specifically, the de novo transcribed miR-430 targets are also controlled post-transcriptionally.

#To Referee 2 Minor points:
1. The authors describe titration of the 4-SU labelling and indicate that using 75 mM embryos develop normally, whereas in 100 mM the embryos arrest at sphere. This is a very small difference in concentration. It is conceivable, that injection volume variation could lead to a proportion of embryos in the pools being affected by receiving toxic levels of the reagent. How did the authors confirm the viability of embryos?
We appreciate the reviewer's concern and understand the importance of ensuring the viability of embryos in our study. To confirm embryo viability, we employed a two-step approach. First, we estimated the injection volume by referencing the volume of a water sphere in a mineral oil droplet under the microscope, a method consistent with previous studies (PMID: 34430313). Second, we assessed embryo viability up to approximately 6 hours post-injection, a time at which WT-non-injected embryos typically reach a developmental stage referred to as the 'shield' stage. Embryos were considered viable if most of them reached this stage at a similar time as WT embryos. Importantly, our monitoring ceased after this time point, and it is plausible that development resumed, with higher doses potentially causing a delay rather than a complete arrest. We hope this additional information clarifies our approach and assures the reviewer of the rigor applied in assessing embryo viability.

2. Large body of literature of ZGA regulation is not reflected by the comparably small number of references cited in this ms.
We appreciate the reviewer's feedback regarding the number of references cited in our manuscript. We understand the importance of referencing relevant literature in providing a comprehensive context for our study. Following the journal's reference guidelines, which limit the number of references that can be cited, we have made efforts to increase the number of papers referenced by approximately 70%. This adjustment was made to accommodate the inclusion of additional previous works that are crucial for the proper refinement of our study’s conclusions. We believe that this update enhances the completeness of our manuscript and better acknowledges the existing body of literature on ZGA regulation.

3. There is need for checking the ms for typos and grammatical errors. Without comprehensive list, some examples are below:
SLAM is written with all caps #This is now corrected in the manuscript.
Line 207: 'miR-430 evidences' - this does not make clear sense. #This is now clarified.
Figure 2D caption: 'Non-labaled'. #This is now changed in the manuscript.
Line 83 'clearer'. #This is now corrected in the manuscript.
Figure 3C: 'Cahange'. #That typo was addressed in the text.
'Replica' should be replaced with 'Replicate' in the Supp. Figures. #This is now corrected in figures.
Font sizes and styles (use of bold) ought to be harmonised for easier reading and interpretation of the densely presented Figures. #This is now corrected in figures.

**Second round of review**

**Reviewer 1**

In this manuscript, Amaral et al. apply metabolic labeling of mRNA to dissect the transcriptional origin of miR-430 targets and the extent of the miRNA regulation. This work nicely complements previous work more focused in uncovering maternal and zygotic genes using the same mRNA labeling approach. Of particular importance is the fact that the authors show how miRNA-mediated repression needs to be factored in when establishing transcriptional rates from SLAM-Seq data.
In this revised version of the manuscript the authors have addressed my comments regarding figure labeling and figure referencing in the main text. In addition, the authors have improved the text for clarity and to convey precisely what is previous knowledge in the field and the new contributions stemming from this work.
The manuscript is ready for publication in Genome Biology and will be of interest to researchers trying to understand the interplay of transcriptional and post-transcriptional dynamics of biological transitions.

**Reviewer 2**

This revised ms by addressed satisfactorily most of my concerns by substantially changing the text to more objectively interpret the novelty of their results. I have a small number of remaining suggestions to make.

1. Line 34: The wording could be improved: ‘SLAM-seq can be used to disentangle transcriptional and posttranscriptional regulation of mRNA levels’ is not precise as their miR-430 KD data they refer to in this sentence is distinguishing post-transsriptional regulation of de novo transcribed genes rather than transcriptional mRNA regulation events.

2. Subchapter: ‘zygotic waves’
I continue to struggle with the suggestion of waves of gene expression claimed in their time course data. Maybe it is because that definition of the ‘wave’ is not clear. To me, the term wave implies some degree of coordinated pattern variation, which is not demonstrated in the plots of Figure 3B. By the way these plots ought to be described in more detail both in the text and legend and preferably given separate panel numbers as the distinction between the two plots is not described well.
In Figure 3B top, we are presented by a suggestion (hypothesis) for an hourly wave profile, suggesting step change in gene expression in an hourly fashion. What I see in the data is that when measured hourly, at every hour there are large number of genes that show substantially increased levels of activity in relation to the previous hour without any particular pattern for a time point. I do not see how this reflects waves. Firstly, the hourly number of genes do not show clear trends, unlike their model analysis of First and Second wave which clearly does. I can easily imagine a scenario, that there is a continuity of increasing number of genes getting switched on without any particular step change at discrete time points (i.e. a straight line in Figure 3B top), but because the measurements are per hour, the accumulation of gene numbers with increased expression appears large and discrete. There is no need (or evidence for) for waves of activation, a gradual increase in gene numbers, say every 10 min, would also arrive to the same plot pattern when measured hourly and as shown in the panels of Figure 3B. Therefore, I suggest that the term ‘waves’ should be removed and replaced with a statement that reflects the only demonstrable effect detected so far, which is the continued nature of gene activation beyond the main wave of ZGA. For the ‘wave’ claim some coordination of gene activation at discrete time points would be necessary to show.

3. Figure 3 presentation could (should) be improved. The gene expression levels per stage do not show the hypothesised wave-like effect. By showing the pattern of gene expression gene by gene would better reflect the expression dynamics observed in the SLAM-seq data and may help in demonstrating coordinated patterns of expression dynamics. This could be heatmaps of CPMs/gene/stage and clustered according to gene expression change in the time course; or the expression change data could be shown by bundled presentation of line graphs of CPMs/gene during their time course and shared colors could be used to distinguish genes activated similarly or coordinately. Such visualisation would potentially reveal true wave-like coordination of gene expression. Unless a wave like effect is clearly shown, the schema in Figure 3B top is not helpful and can be misleading.

Minor comments:
SLAMseq (or SLAM-seq) should be all capital letters not just first capital.
L30: miR-430
L36: ‘such valuable’: avoid subjective statement

**Authors’ response to reviewers**

Reviewer #1 reports:
In this manuscript, Amaral et al. apply metabolic labeling of mRNA to dissect the transcriptional origin of miR-430 targets and the extent of the miRNA regulation. This work nicely complements previous work more focused in uncovering maternal and zygotic genes using the same mRNA labeling approach. Of particular importance is the fact that the authors show how miRNA-mediated repression needs to be factored in when establishing transcriptional rates from SLAM-Seq data.
In this revised version of the manuscript the authors have addressed my comments regarding figure labeling and figure referencing in the main text. In addition, the authors have improved the text for clarity and to convey precisely what is previous knowledge in the field and the new contributions stemming from this work.
The manuscript is ready for publication in Genome Biology and will be of interest to researchers trying to understand the interplay of transcriptional and post-transcriptional dynamics of biological transitions.

Authors’ Comments: We appreciate your comments and feedback, we believe they made our manuscript better than before.

Reviewer #2 reports:
This revised ms by addressed satisfactorily most of my concerns by substantially changing the text to more objectively interpret the novelty of their results. I have a small number of remaining suggestions to make.
Authors’ Comments: We again thank you for your time and suggestions.

1. Line 34: The wording could be improved: ‘SLAM-seq can be used to disentangle transcriptional and posttranscriptional regulation of mRNA levels’ is not precise as their miR-430 KD data they refer to in this sentence is distinguishing post-transsriptional regulation of de novo transcribed genes rather than transcriptional mRNA regulation events.
Authors’ Comments: To improve precision, we have now deleted this comment so it is not linked to the previous sentence.

2. Subchapter: ‘zygotic waves’
I continue to struggle with the suggestion of waves of gene expression claimed in their time course data. Maybe it is because that definition of the ‘wave’ is not clear. To me, the term wave implies some degree of coordinated pattern variation, which is not demonstrated in the plots of Figure 3B. By the way these plots ought to be described in more detail both in the text and legend and preferably given separate panel numbers as the distinction between the two plots is not described well.
In Figure 3B top, we are presented by a suggestion (hypothesis) for an hourly wave profile, suggesting step change in gene expression in an hourly fashion. What I see in the data is that when measured hourly, at every hour there are large number of genes that show substantially increased levels of activity in relation to the previous hour without any particular pattern for a time point. I do not see how this reflects waves. Firstly, the hourly number of genes do not show clear trends, unlike their model analysis of First and Second wave which clearly does. I can easily imagine a scenario, that there is a continuity of increasing number of genes getting switched on without any particular step change at discrete time points (i.e. a straight line in Figure 3B top), but because the measurements are per hour, the accumulation of gene numbers with increased expression appears large and discrete. There is no need (or evidence for) for waves of activation, a gradual increase in gene numbers, say every 10 min, would also arrive to the same plot pattern when measured hourly and as shown in the panels of Figure 3B. Therefore, I suggest that the term ‘waves’ should be removed and replaced with a statement that reflects the only demonstrable effect detected so far, which is the continued nature of gene activation beyond the main wave of ZGA. For the ‘wave’ claim some coordination of gene activation at discrete time points would be necessary to show.
Authors’ Comments: We acknowledge the reviewer point of view and understand that sampling frequency can interfere with interpretation of results. We’ve made previous efforts in addressing this in our discussion section. We appreciate reviewer’s patience and further clarification, and now we modified it the discussion even further to make sure we cover this point. Additionally, we have no intention of claiming and a coordinated cascade-like onset of ZGA (something similar to Lee et al., 2013, PMID: 24056933), and now we understand what the Reviewer is thinking about waves, therefore we have removed the term “wave” when referring to our analysis, to prevent any confusion regarding its interpretation. We have replaced it with “hourly detected groups of transcriptional activation events” or similar wording. We believe these changes will reflect more our analysis results for this figure, which are the initial detection of transcriptional events by labeled read counts in every given hour.

3. Figure 3 presentation could (should) be improved. The gene expression levels per stage do not show the hypothesised wave-like effect. By showing the pattern of gene expression gene by gene would better reflect the expression dynamics observed in the SLAM-seq data and may help in demonstrating coordinated patterns of expression dynamics. This could be heatmaps of CPMs/gene/stage and clustered according to gene expression change in the time course; or the expression change data could be shown by bundled presentation of line graphs of CPMs/gene during their time course and shared colors could be used to distinguish genes activated similarly or coordinately. Such visualisation would potentially reveal true wave-like coordination of gene expression. Unless a wave like effect is clearly shown, the schema in Figure 3B top is not helpful and can be misleading.
Authors’ Comments: We understand the reviewer concern on our hypothesis graph and interpretation. Our intent with Fig3B is to look at the initial onset of transcription per gene given our resolution, not follow their trajectories overtime. We agree that improvements on that figure would be beneficial, so we modified Fig3B top to make sure it aligns with our goal of identify the initially detection of the newly transcribed mRNA regardless of when in-between timepoints transcription starts for it and to prevent confusion about one group genes (previously wave) regulating the next now as a cascade or domino effect. Moreover, we have previously plotted the heatmap that the reviewer suggested, but we think it will create more confusion because that a heatmap is interesting to start dissecting the trajectories of particular genes or smaller clusters, but that is not the goal of this work. In the future, and as we have discussed it in the paper, it will be very interesting to do this type of plots after knocking down particular genes (e.g. transcription factors) to identified which are the genes and potential network affected by the absence of a particular regulator.

Minor comments:
SLAMseq (or SLAM-seq) should be all capital letters not just first capital. #This has been corrected.
L30: miR-430 #This has been corrected.
L36: ‘such valuable’: avoid subjective statement #This has been corrected.
